# Genetic Modeling of Lysosomal Storage Disorders (LSDs) in the Brain–Midgut Axis of *Drosophila melanogaster* During Aging

**DOI:** 10.3390/cells15010006

**Published:** 2025-12-19

**Authors:** Sophia P. Markaki, Nikole M. Kiose, Zoi A. Charitopoulou, Stylianos Kougioumtzoglou, Athanassios D. Velentzas, Dimitrios J. Stravopodis

**Affiliations:** Section of Cell Biology and Biophysics, Department of Biology, School of Science, National and Kapodistrian University of Athens (NKUA), 157 01 Athens, Greece; smarkak@biol.uoa.gr (S.P.M.); nikoletajoanaq@gmail.com (N.M.K.); zoecharitopoulou@gmail.com (Z.A.C.); steliosk7777@gmail.com (S.K.)

**Keywords:** aging, brain, *Drosophila melanogaster*, Fabry disease, GAL4/UAS, Gaucher disease, Hunter syndrome, Hurler syndrome, midgut, Niemann–Pick disease, Pompe disease, RNAi, Sly disease, Tay–Sachs/Sandhoff disease(s)

## Abstract

Lysosomal storage disorders (LSDs) are a group of rare inherited diseases caused by mutations in the genes encoding the proteins involved in normal lysosomal functions, leading to an accumulation of undegraded substrates within lysosomes. Among the most prominent clinical features are neurological impairment and neurodegeneration, arising from widespread cellular dysfunction. The development of powerful and reliable animal model systems that can in vivo recapitulate human LSD pathologies is critical for understanding disease mechanisms and advancing therapeutic strategies. In this study, we identified the *Drosophila melanogaster* orthologs of human LSD-related genes using the DIOPT tool and performed tissue-specific gene silencing along the brain–midgut axis via the use of GAL4/UAS and RNAi combined technologies. Transgenic fly models presented key features of human LSD pathologies, including significantly shortened lifespans and a progressive locomotor decline that serves as a measure for neuromuscular disintegration, following age- and sex-dependent patterns. These phenotypic parallels in pathology strongly support the functional relevance of the selected orthologs and underscore the value of *Drosophila* as a versatile in vivo model system for advanced LSD pathology research, offering state-of-the-art genetic tools for molecularly dissecting disease mechanisms and providing cutting-edge novel platforms for high-throughput genetic and/or pharmacological screening, moving towards development of new therapeutically beneficial drug-based regimens and mutant gene-rescue schemes.

## 1. Introduction

Lysosomal storage disorders (LSDs) are a group of rare diseases comprising more than 70 inherited metabolic disorders that are characterized by lysosomal dysfunction and a subsequent accumulation of undegraded substrates within lysosomes. LSDs are monogenic diseases caused by alterations in the genes encoding the proteins involved in normal lysosomal function, such as lysosomal enzymes and lysosomal membrane proteins, and their combined prevalence is estimated to be approximately 1 in 8000 live births [1,2,3,4].

Lysosomal impairment leads to the dysregulation of a diverse range of cellular processes associated with lysosomes, such as membrane repair, vesicle trafficking, lipid homeostasis, signaling, cell death pathways, autophagic flux, and the clearance of autophagosomes. Therefore, autophagic impairment has been described as a common mechanism of pathology in an increasing number of LSDs [4,5,6]. Despite their heterogeneity, their major clinical symptoms include hepatosplenomegaly, pulmonary and cardiac disorders, skeletal abnormalities, and, often, central nervous system (CNS) dysfunction, with patients frequently being presented with progressive neurodegenerative clinical courses [6,7]. Typically, LSDs are primarily classified according to the biochemical properties of the accumulated undegraded substrate and these include sphingolipidoses, glycogen storage diseases, and mucopolysaccharidoses [4,8].

Sphingolipidoses are disorders caused by genetic defects in the catabolism of sphingosine-containing lipids and their accumulation affects both the CNS and peripheral organs [9]. Gaucher, Fabry, Tay–Sachs, and Niemann–Pick are classified among the most common sphingolipid metabolism diseases [8,9,10]. Gaucher disease (GD), which is subdivided into three different types, is the most prevalent form of sphingolipidoses and is caused by mutations in the *GBA* gene, which encodes for the lysosomal hydrolase β-Glucocerebrosidase, responsible for the degradation of glucosylceramide into glucose and ceramide [4,8,9,10]. Fabry, an inherited X-linked disease, is the second most common form of sphingolipidoses [2] and is caused by mutations in the *GLA* gene encoding the enzyme α-Galactosidase A, which catalyzes the lysosomal hydrolysis of globotriaosylceramide [4,7,10]. Tay–Sachs, a type of GM2 gangliosidosis, presents with severe neurological symptoms and is caused by mutations in the *HEXA* gene encoding the enzyme β-Hexosaminidase A, which is responsible for breaking down GM2 gangliosides, resulting in their toxic accumulation in neuronal tissues [11]. Niemann–Pick is a group of predominantly neurodegenerative disorders classified in types A and B, caused by mutations in the *SMPD1* gene, while type C derives from mutations in the *NPC1* or *NPC2* genes. In types A and B, the affected enzyme is Sphingomyelinase (ASM), leading to sphingomyelin buildup, whereas, in Niemann–Pick type C, proteins that mediate cholesterol transport from endosomes/lysosomes are seriously affected, causing endo-lysosomal accumulation of cholesterol, glycosphingolipids, and sphingomyelin, resulting in severe neurological pathology [1,6,12].

Glycogen storage diseases (GSDs) comprise a group of inherited metabolic disorders caused by mutations in the genes encoding the enzymes of glycogen metabolism. Among them, Pompe disease, also known as GSD II, is classified as a major LSD family member. Pompe disease results from mutations in the *GAA* gene encoding α-Glucosidase, which is a key lysosomal enzyme responsible for the hydrolysis of glycogen to glucose. The hallmark of Pompe disease is glycogen accumulation in lysosomes, predominantly in muscle cells, leading to cardiorespiratory failure [4,7,8].

Mucopolysaccharidoses (MPSs) form a group of eleven LSD pathologies, characterized by the cellular accumulation of glycosaminoglycans (GAGs), which are negatively charged polysaccharides essential for several cellular processes, including signaling and development. The classification of MPSs is based on mutations in specific enzymes that catabolize target substrates, with MPS I, II, and III being the most common ones [4]. MPS type I (MPS I) is caused by the deficiency of lysosomal hydrolase α-L-Iduronidase (IDUA), leading to the accumulation of dermatan- and heparan-sulfate inside lysosomes of a wide range of tissues. The severe form of MPS I, known as Hurler syndrome, is characterized by early-onset progressive somatic and neurological impairments [13]. MPS II, also known as Hunter syndrome, is caused by mutations in the *IDS* gene on the X chromosome and is typically described by neurological deterioration. These mutations result in a critical deficiency of Iduronate-2-sulfatase (IDS), an enzyme responsible for breaking down dermatan- and heparan-sulfate. Finally, Sly disease, also known as MPS VII, is caused by mutations in the *GUSB* gene, resulting in β-Glucuronidase (GUSB) enzyme deficiency. This leads to the accumulation of dermatan-, heparan-, and chondroitin-sulfate GAGs, causing progressive multi-system dysfunctions [4,14].

Neurological dysfunction and progressive neurodegeneration are key symptoms of LSDs [6]. The study of animal model organisms is imperative for advancing our understanding of human pathologies, thus enabling the identification of novel disease-related pathways that have the potential to serve as drug targets. Furthermore, recent progress has led to the development of more powerful and reliable animal models that can more precisely mirror aberrant phenotypes and the pathological processes of human diseases and, in particular, LSDs [15,16,17]. A recently explored therapeutic approach for LSDs is a targeted gene therapy that uses genome-editing technologies, like CRISPR/Cas9. However, these strategies encounter several technical challenges and bioethical considerations, making it essential to study their effects in vivo, using animal models that can closely replicate LSD-specific phenotypes. To thoroughly evaluate the mechanisms of LSD progression, mammalian genetic models have proven invaluable for characterizing novel pathways and validating potential therapies [18,19]. Complementing these studies, non-mammalian vertebrate models, such as zebrafish, with more than 60 identified LSD lines, have also contributed significantly to our understanding of lysosomal biology and drug discovery [17]. However, despite these advances, the complexity and cost of vertebrate models often limit their utility for large-scale, high-throughput genetic and pharmacological screenings, underscoring the necessity for robust invertebrate systems like *Drosophila melanogaster*.

Given the imminent need for robust in vivo models, we have herein generated transgenic *Drosophila* flies, by utilizing the binary GAL4/UAS and RNAi gene-targeting technology, to mechanistically illuminate LSD-associated pathologies during aging. This platform provides a dynamic and versatile tool to deeply investigate systemic pathologies and successfully explore novel therapies for LSDs.

## 2. Materials and Methods

### 2.1. Ortholog Prediction and Definition of DIOPT Score and Homology Ranking

To identify *Drosophila* orthologs of human LSD-related genes, we used the DRSC (*Drosophila* RNAi Screening Center, Boston, MA, USA) Integrative Ortholog Prediction Tool (DIOPT) [20]. The DIOPT integrates results of multiple ortholog-mapping tools based on different algorithms and calculates a numeric score reflecting the number of tools that support a given ortholog gene–pair relationship. In version 9, the numeric DIOPT score for fly–human gene pairs ranges from 1 to 19 (maximum = 19). The DIOPT also provides ortholog ranks (high, moderate, and low) that reflect the confidence level of each predicted orthology relationship between genes of different species. The rank is assigned from a combination of the numeric DIOPT score and the “best score” flag that indicates whether the pair is the top match in forward (fly to human) and/or reverse (human to fly) searches [20,21,22]. Specifically, high rank pairs are the best in both forward and reverse (reciprocal best) searches and have a DIOPT score ≥ 2, moderate rank pairs are the best in either forward or reverse searches and have a DIOPT score ≥ 2 or a DIOPT score ≥ 4, and low rank pairs encompass all other cases [22]. Because rank depends on both the numeric score and best-hit status, it cannot be mapped to simple DIOPT-score ranges alone.

### 2.2. Structural Alignment

For the structural alignment of *Homo sapiens* (human) and *Drosophila* (fly) LSD-related proteins, AlphaFold-specific molecular models were obtained from the neural network-based AlphaFold2 Protein Structure Database [23,24,25] and the generated protein models were structurally aligned with the PyMOL (v3.1) molecular graphics system [26]. The Root Mean Square Difference (RMSD) value of each alignment was used as an indicator for the reliability of the structural alignment [27].

### 2.3. Drosophila melanogaster Strain Stocks and Culturing Conditions

The *Drosophila melanogaster* transgenic RNAi fly strains utilized in this study are listed in Table 1. These stocks, along with the pan-neuronal driver elav-GAL4 (genotype: w[*]; P{w[+mC] = GAL4-elav.L}3; RRID:BDSC_8760), were obtained from the Bloomington *Drosophila* Stock Center (NIH P40OD018537, Bloomington, IN, USA). The *D. melanogaster* midgut-specific NP1-GAL4 driver strain was kindly provided by Dr. Eric H. Baehrecke [28] (Department of Cancer Biology, University of Massachusetts, Medical School, Worcester, MA, USA).

All fly stocks were maintained at 25 °C, in a relative humidity of 55–65%, under a 12 h light/dark photoperiod and using a laboratory standard *Drosophila* nutrition medium (6.4% rice flour, 5% tomato paste, 3.2% sugar, 0.8% yeast, 0.8% agar, 0.13% Tegosept, 0.4% ethanol, and 0.4% propionic acid).

### 2.4. RNA Extraction and RT-qPCR

Isolation of total cellular RNA from RNAi-targeted fly heads was performed using the PureLink™ RNA Mini Kit (Invitrogen, Thermo Fisher Scientific, Waltham, MA, USA), according to the manufacturer’s instructions. The concentration and quality of the isolated RNA were determined using the NanoDrop One UV-Vis. spectrophotometer (Thermo Fisher Scientific, Waltham, MA, USA). First-strand cDNA was synthesized using the SuperScript™ IV First-Strand Synthesis System (Invitrogen, Thermo Fisher Scientific, Waltham, MA, USA), following the manufacturer’s protocol.

Relative expression of the herein studied genes was examined by Reverse Transcription (real-time) quantitative Polymerase Chain Reaction (RT-qPCR), using specific primers (Table S1), the Fast SYBR^®^ Green Master Mix (Applied Biosystems, Thermo Fisher Scientific, Waltham, MA, USA), and the Applied Biosystems StepOne^™^ (real-time) qPCR System (Thermo Fisher Scientific, Waltham, MA, USA), as described by the manufacturer’s guidelines. As a suitable internal control for normalization of gene expression values, the housekeeping gene *Actin 5C* was used accordingly. To ensure reproducibility, each assay was performed in technical triplicates, while three negative controls were also included in the analysis. Fold reductions in transcript levels were determined using the comparative 2^−ΔΔCt^ method [29], which calculates changes in gene expression as a relative fold difference between the gene(s) of interest and the reference gene. Results were presented as a percentage of the relative gene reduction in RNAi-targeted [specifically in neuronal (brain) tissues] flies compared to control populations. Each experiment was performed three different times using independent genetic crosses.

### 2.5. Longevity Measurement

To study viability, newly eclosed (0–24 h) transgenic flies from each fly cross were collected and allowed to mate to ensure physiological maturity. Subsequently, flies were separated by sex and maintained in vials at a controlled density (~20–25 flies per vial). This once-mated protocol ensures that females are physiologically mature, while avoiding the effects of continuous male harassment or egg retention stress associated with enforced virginity [30,31]. Furthermore, mating triggers essential endocrine remodeling of the midgut, leading to the activation of the lipid metabolism genes required for adult homeostasis [32]. Flies were maintained in a constant temperature and humidity chamber throughout the experimental period and transferred to fresh food every 3 days. Survival curves were generated by daily counting of the deceased flies. For each viability experiment, a minimum sample size of *n* = 100 flies per sex and genotype was employed to ensure statistical significance. All viability experiments for control and RNAi-expressing strains were performed at the same time and under identical conditions. Each experiment was performed in at least 3 independent biological replicates.

### 2.6. Negative Geotaxis Assay

The locomotor performance of RNAi-targeted flies was quantified using the negative geotaxis (climbing) assay. Flies of both sexes were initially kept together and before the experimental procedure they were anesthetized and divided into male and female populations (groups of ~20–25 flies each). Each experimental group was then placed in an empty 100 mL cylinder, with a boundary line drawn at the 60 mL mark (10 cm height), and the flies were allowed for 1 min to acclimatize to the environment. To ensure they climbed simultaneously, flies were gently tapped to the bottom of the cylinder. After a time interval of 20 s, the number of flies that reached or exceeded the 60 mL limit was counted. Five trials with a 1 min time interval were performed for each group. The same populations were tested at different ages, excluding flies that died or flew away. Control and RNAi-targeted fly groups were examined simultaneously. Total sample size for each fly cross and gender was set at (a minimum of) 100 flies. Three independent biological replicates were used for each fly group examined.

### 2.7. Statistical Analysis

Statistical analysis and graphical presentation of the obtained results were performed using the Statistical Package for Social Sciences (IBM SPSS v25.0 for Windows IBM Corp., New York, NY, USA). The data from the longevity experiments were analyzed with the Kaplan–Meier survival test, using the Log Rank, Breslow test, and Τarone–Ware statistical packages. Climbing graphs were plotted as the mean pass rate per genotype/timepoint with the Sample Standard Deviation (±SSD) value. Statistically significant differences between the compared genotypes, at each timepoint, were evaluated by the independent *t*-test analysis. Significance was accepted at the following levels: *p* < 0.05 (*), *p* < 0.01 (**), and *p* < 0.001 (***).

## 3. Results

### 3.1. From Humans to Flies: Identification of LSD-Related Gene Orthologs Using the DIOPT

The first step for studying human disease genes in animal model organisms such as *Drosophila* is to recognize the putative orthologs that are being associated with the respective diseases. Ortholog genes facilitate functional genomics by allowing hypotheses concerning the functions of genes in one species to be deduced from their orthologs in another species. In Table 2, we present an extensive DIOPT-derived list, corresponding to nearly all human LSD-related genes, along with their putative orthologs in *Drosophila*. In addition, we provide the DIOPT rank and the associated RNAi strains available from the Bloomington (BDSC) and Vienna (VDRC) [33] *Drosophila* Stock/Resource Centers, thus offering a useful resource for designing genetic studies on evolutionary conserved disease genes.

Following ortholog prediction, we focused on screening LSDs not previously studied in *Drosophila* or examined using alternative genetic tools. Our overall objective is to identify novel genotype–phenotype associations between humans and *Drosophila*, to advance our understanding of the molecular mechanisms underlying LSDs. To investigate the systemic functional importance of LSD-related genes in vivo, we performed targeted gene knockdown of their *Drosophila* orthologs using the GAL4/UAS genetic system combined with RNAi technology [33,34]. Gene silencing was carried out in a tissue-specific manner, targeting the brain (elav-GAL4) and the midgut (NP1-GAL4). We specifically targeted the midgut because the integrity of the intestinal epithelium is an essential determinant of organismal health and viability. In diverse species, intestinal barrier dysfunction is a shared pathophysiological hallmark of aging and in *Drosophila* it has been causally linked to systemic metabolic dysregulation and inflammation [35,36]. Crucially, the adult *Drosophila* midgut is a highly sexually dimorphic organ, displaying extensive sex differences in the expression of genes governing growth and metabolism. Specifically, female midguts exhibit high plasticity and lipid turnover to support reproduction, whereas male midguts maintain a more static homeostatic profile [37,38,39].

To quantify gene-function aberrations, we adopted a phenotype-driven hierarchical screening strategy. First, life expectancy was assessed for all candidates in both tissue types. Subsequently, locomotor impairment was specifically evaluated in those *Drosophila* models that exhibited the most pronounced reductions in lifespan (particularly where effects were observed in both tissues). This secondary behavioral screening focused on the neuronal (elav) knockdown, as negative geotaxis (climbing activity) is a widely used method for evaluating neuromuscular dysfunction resulting from neuron-specific gene silencing [40]. For all LSDs herein examined, the predicted *Drosophila* orthologs being selected for further investigation were characterized by high or moderate DIOPT rankings (Table 2).

### 3.2. Structural Conservation of LSD-Associated Proteins Between Homo Sapiens and Drosophila melanogaster

Three-dimensional structural alignment via PyMOL [26] is a valuable tool for revealing structural conservation between ortholog proteins with high sequence similarity and for gaining insights into their structural characteristics. Initially, all selected LSD-related structures of human and *Drosophila* proteins were retrieved from the neural network-based protein-structure prediction tool AlphaFold2 [23,24,25]. Next, PyMOL alignment was employed, with sequence alignment being followed by structural superposition and subsequent minimization of the Root Mean Square Deviation (RMSD) between the aligned residues through the execution of refinement cycles to reject structural outliers identified during alignment. RMSD value between corresponding atoms of two protein chains is the most common estimator of structural similarity, with an RMSD below 3 Å typically indicating close homology [27]. Interestingly, protein comparisons of all of the *Drosophila* orthologs derived from the DIOPT that carried high or moderate homologies to their human LSD-related counterparts yielded low RMSD values, ranging from 0.454 to 1.376 Å, with the majority being measured below 1 Å (Figures S1–S8), thus indicating minor structural variation. Only the comparison of protein structures produced from the human gene *GAA* (implicated in Pompe disease) and its *Drosophila* ortholog *tobi* (Figure S5), which shows low homology in DIOPT, resulted in a high RMSD value of 3.291 Å, strongly suggesting a comparatively greater disparity between the human and fly respective gene products. For all the alignments herein examined, each *Drosophila* protein structure was aligned to (superimposed with) its respective human reference structure. The high degree of structural similarity observed between the aligned (superimposed) ortholog proteins not only reflects their evolutionarily conserved architecture, but also indicates conserved functional properties and roles, thereby strongly supporting the validity of using *Drosophila* as a powerful, versatile, and multifaceted animal model system for in vivo investigation of human LSD-linked pathologies.

### 3.3. Modeling of Sphingolipidoses in Drosophila

#### 3.3.1. Gaucher Disease

**Figure 1:**
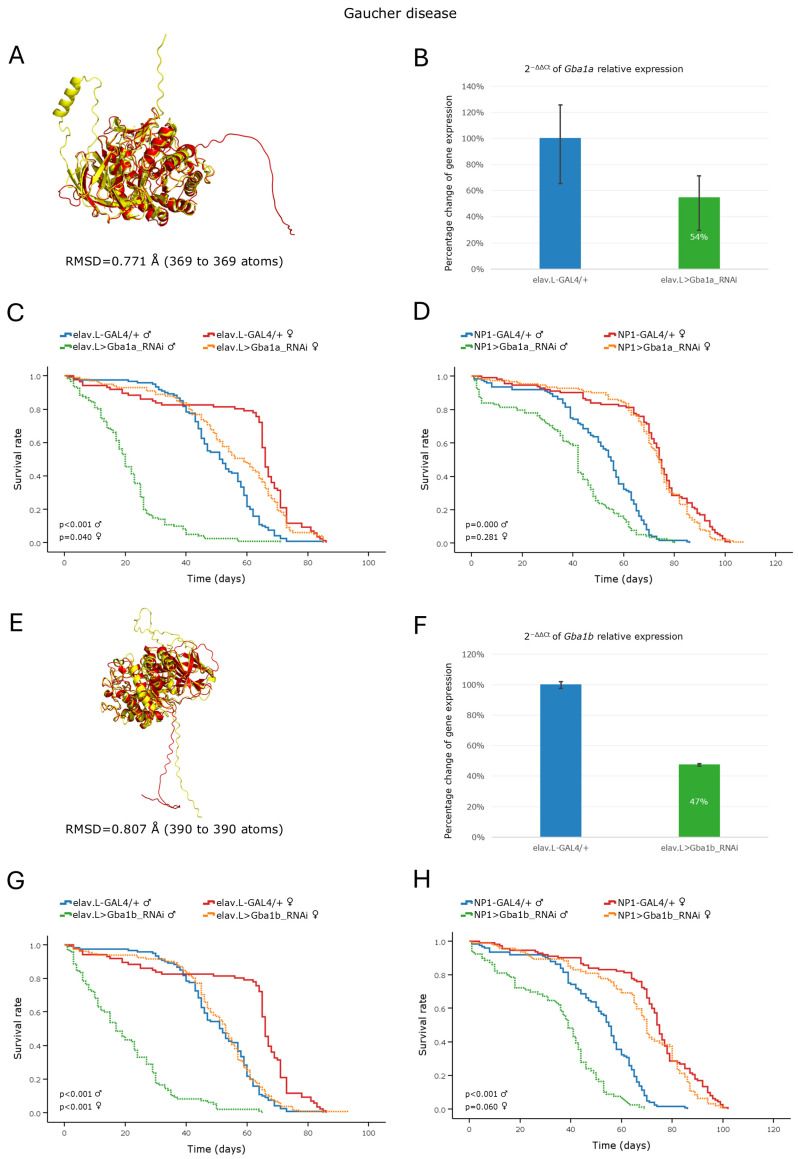
In vivo genetic modeling of Gaucher disease in *Drosophila*, via *Gba1a* and *Gba1b* ortholog gene targeting specifically within the brain–midgut axis. (**A**) Structural alignment of the AlphaFold-predicted protein structures that are encoded by the human *GBA1* gene (red) and its *Drosophila* ortholog *Gba1a* (yellow). The human protein structure was aligned to the respective *Drosophila* structure using the PyMOL molecular graphics system. (**B**) Relative expression analysis of the *Gba1a* gene in fly neuronal (brain) tissues following its (*Gba1a*) RNAi-mediated knockdown (elav.L > Gba1a_RNAi), compared to control flies (elav.L-GAL4/+), as determined by RT-qPCR. (**C**) Lifespan profiling of male and female flies following *Gba1a* gene knockdown specifically within the nervous system (brain). (**D**) Survival curves of male and female flies being subjected to *Gba1a* gene downregulation that specifically targets midgut tissues (NP1 > Gba1a_RNAi). (**E**) Structural alignment of AlphaFold-derived protein structures that are encoded by the human *GBA1* gene (red) and its *Drosophila* ortholog *Gba1b* gene (yellow), with the human reference protein being aligned to the *Drosophila* one through employment of the PyMOL molecular graphics system. (**F**) Relative expression levels of the *Gba1b* gene in neuronal (brain) tissues of RNAi-targeted flies (elav.L > Gba1b_RNAi), compared to control flies (elav.L-GAL4/+), assessed by RT-qPCR. (**G**) Survival curves of male and female flies following *Gba1b* gene knockdown specifically targeting the nervous system (brain). (**H**) Lifespan profiles of male and female flies after *Gba1b* gene silencing that specifically targets midgut tissues (NP1 > Gba1b_RNAi). Sample sizes: a total of *n* > 300 flies per genotype and sex (pooled from three independent biological replicates, with *n* > 100 per replicate).

Sphingolipidoses constitute an essential group of LSDs that are characterized by the accumulation of sphingolipids [10]. Among them, Gaucher disease is the most common one [2] and is distinguished by its three different subtypes, all caused by mutations in the *GBA1* gene [4]. A DIOPT analysis to identify *Drosophila melanogaster* orthologs revealed two candidate genes, *Gba1a* and *Gba1b*, both exhibiting high homology scores (Table 2) and encoding protein products with strong structural similarities to their human counterparts (Figure 1A,E, for *Gba1a* and *Gba1b*, respectively). Therefore, we, next, proceeded to investigate the tissue-specific, RNAi-mediated, downregulation of both *Drosophila* orthologs along the brain–midgut axis. To validate the efficiency of gene silencing, and thus the reliability of our genetic models, we quantified *Gba1a* and *Gba1b* gene expression levels following neuronal-specific knockdown. The analysis revealed a significant reduction in mRNA expression of ~46% for *Gba1a* (Figure 1B) and ~53% for *Gba1b* (Figure 1F), relative to the control fly brains.

RNAi-mediated downregulation of the *Gba1a* or *Gba1b* gene that specifically targets the nervous system (brain) caused a severe decrease in male fly life expectancy, with a median reduction in survival of ~31 and ~34 days, respectively, compared to control male flies (Figure 1C,G). Although female flies also exhibited a reduced median survival in response to neuronal-specific (brain) knockdown of either gene, their pathogenic effects were notably less pronounced compared to male ones (Figure 1C,G). Regarding midgut-specific knockdown models, only male flies demonstrated significantly decreased survival rates (Figure 1D,H), with the obtained pathology being comparatively more severe in the *Gba1b*-specific, RNAi-targeted flies.

Furthermore, compared to control flies, adult males with either a *Gba1a* or *Gba1b* gene knockdown profile specifically targeting the nervous system (brain), displayed progressive, age-dependent, locomotor defects (Figure S9), which comparatively proved more detrimental after 10 days of age, post-eclosion. Of note is that due to the high mortality levels of male *Gba1a*- and *Gba1b*-targeted (via RNAi) flies, specifically targeting neuronal tissues (brain), we were unable to collect statistically sufficient numbers of fly individuals for reliably conducting climbing experiments at the 30th day of age, post-eclosion. In contrast, female flies with neuronal-specific (brain) knockdown of either gene retained relatively normal climbing performance compared to their control counterparts (Figure S9).

Taken together, the RNAi-mediated knockdown of either the *Gba1a* or *Gba1b Drosophila* orthologs of the human *GBA1* gene proved able to strikingly recapitulate key pathogenic features of Gaucher disease, strongly supporting the in vivo power and value of the fly brain–midgut axis for reliably modeling and illuminating disease mechanisms and promptly discovering therapeutic regimens.

#### 3.3.2. Fabry Disease

**Figure 2:**
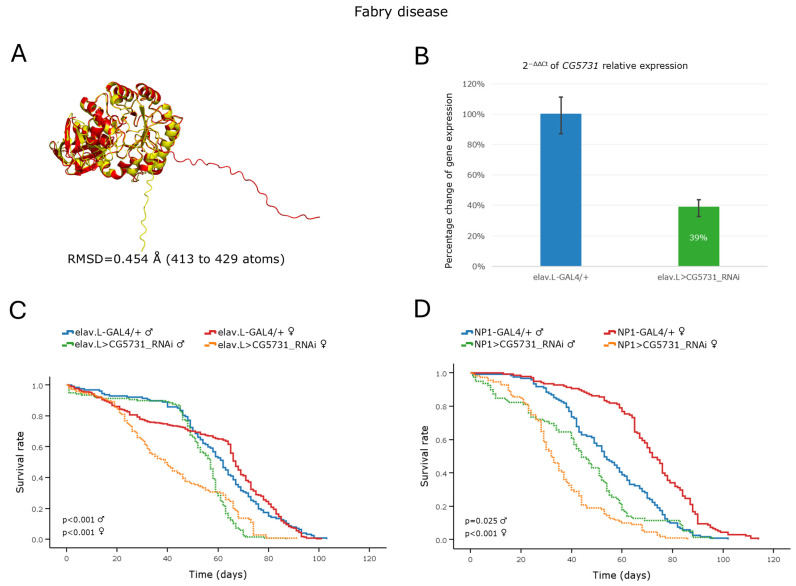
In vivo genetic modeling of Fabry disease in *Drosophila*, via RNAi-mediated targeting of the *CG5731* ortholog gene in the brain–midgut axis during aging. (**A**) PyMOL-mediated structural alignment of AlphaFold-derived protein structures of human *GLA* (red) and its *Drosophila* ortholog *CG5731* (yellow). The human reference sequence (GLA) is aligned to the *Drosophila* protein (CG5731). (**B**) Quantitative analysis of *CG5731* mRNA levels in neuronal tissues of *CG5731*^RNAi^-expressing flies (elav.L > CG5731_RNAi), relative to control (elav.L-GAL4/+), using RT-qPCR. (**C**) Survival curves of male and female flies, following nervous system-specific silencing of the *CG5731* gene. (**D**) Viability profiles of flies characterized by targeted *CG5731* knockdown specifically within midgut tissues (NP1 > CG5731_RNAi) (compared to control). Sample sizes: a total of *n* > 300 flies per genotype and sex (pooled from three independent biological replicates, with *n* > 100 per replicate).

Fabry disease is an X-linked monogenic disorder and it has been reported as the second most common LSD [10]. In humans, the disease is caused by mutations in the *GLA* gene, which encodes the Lysosomal α-Galactosidase A, an enzyme responsible for the hydrolysis of globotriaosylceramide. In *Drosophila*, two orthologs (*CG7997* and *CG5731*) of the human *GLA* gene have been identified. Although both genes receive moderate rankings and modest DIOPT scores (Table 2), the structural comparisons of their cognate protein products (CG7997 and CG5731) to the human counterpart (GLA) reveal very low RMSD values (below 0.5 Å; Figure S10A and Figure 2A), thus indicating the high degree of structural conservation (during species evolution). RNAi-mediated, neuronal (brain)-specific, knockdown of the *CG7997* and *CG5731* genes led to a decrease in mRNA expression of ~8% and ~61%, respectively, compared to that of the control flies (Figure S10B and Figure 2B). As expected from the low knockdown efficiency of *CG7997*, the lifespan of flies that were subjected to RNAi-mediated silencing of this gene along the brain–midgut axis did not significantly differ from that of the control fly population (Figure S10C,D). In contrast, knockdown of *CG5731*, in either the nervous system (brain) or midgut tissues, resulted in flies with a significantly shortened life expectancy (Figure 2C,D). Interestingly, female flies were more severely affected, exhibiting a median lifespan reduction of ~28 days following neuronal (brain)-specific knockdown and of ~40 days after midgut-specific silencing of the *CG5731* gene (Figure 2C,D).

Furthermore, while flies of both sexes presented locomotor deficiencies as early as day 10 post-eclosion, after neuronal-specific knockdown of the *CG5731* gene, as demonstrated by the negative-geotaxis assay (Figure S11), this phenotype was sexually dimorphic, with males exhibiting significant deficits primarily during early adulthood (up to day 15), whereas females displayed a more progressive decline. The observation that female flies, compared to male populations, are more severely affected by *CG5731* downregulation may be mechanistically associated with differences in sex-dependent metabolic demand and hormonal regulation, and/or sexually dimorphic tissue-specific gene expression patterns.

#### 3.3.3. Niemann–Pick Disease

**Figure 3:**
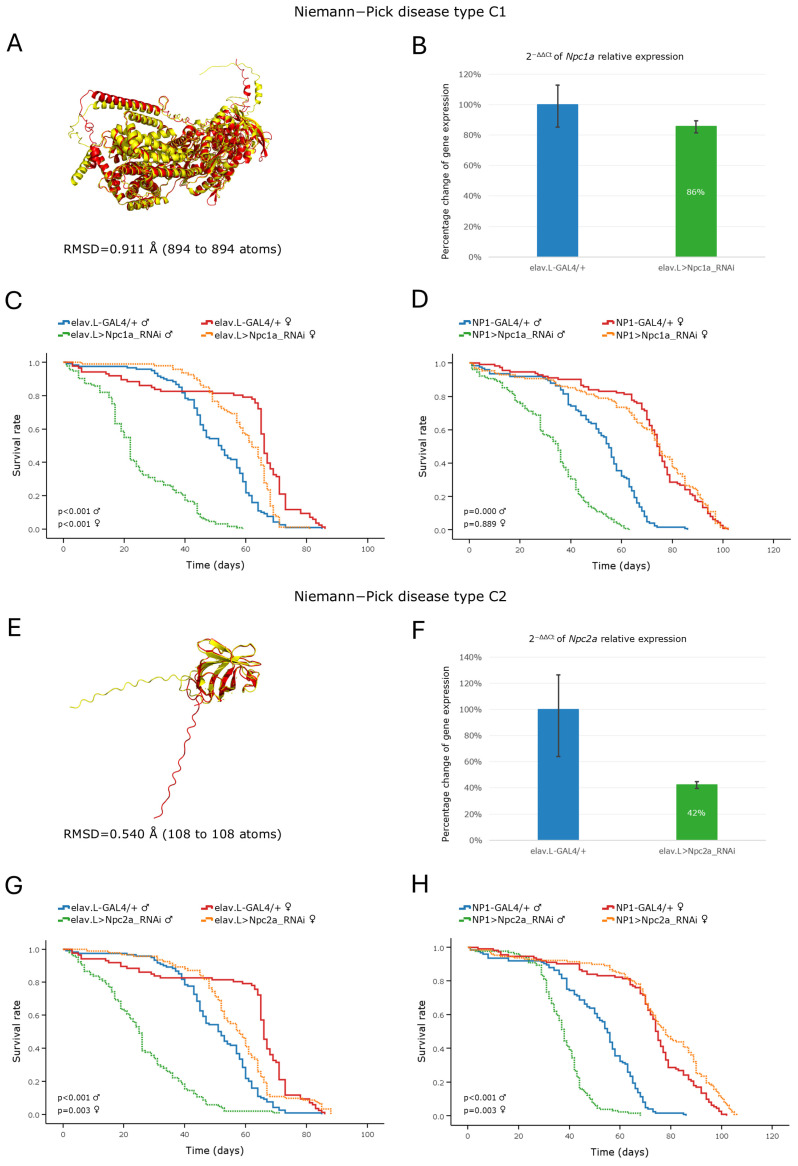
In vivo genetic modeling of Niemann–Pick disease type C in the *Drosophila* brain–midgut axis. (**A**) Structural alignment of AlphaFold-derived protein structures of the human *NPC1* (red) and its *Drosophila* ortholog *Npc1a* (yellow) genes that are generated and visualized via use of the PyMOL molecular graphics system. (**B**) Relative expression levels of the *Npc1a* gene in neuronal tissues of RNAi-targeted flies (elav.L > Npc1a_RNAi) compared to control populations (elav.L-GAL4/+), quantified by RT-qPCR. (**C**) Kaplan–Meier survival curves of male and female flies following neuronal-specific (elav-GAL4) knockdown of *Npc1a*. (**D**) Lifespan profiles of flies that are typified by midgut-specific *Npc1a* gene silencing (NP1 > Npc1a_RNAi). (**E**) Structural alignment of the protein encoded by the human *NPC2* gene (red) and the protein encoded by its *Drosophila* ortholog *Npc2a* (yellow). (**F**) Relative expression of the *Npc2a* gene in neuronal tissues of RNAi-targeted flies (elav.L > Npc2a_RNAi), versus control populations (elav.L-GAL4/+), as examined and quantified by RT-qPCR. (**G**) Kaplan–Meier survival curves following neuronal-specific knockdown of *Npc2a*. (**H**) Survival profiles following midgut-specific knockdown of the *Npc2a* gene (NP1 > Npc2a_RNAi) compared to that of the controls (NP1-GAL4/+). Sample sizes: a total of *n* > 300 flies per genotype and sex (pooled from 3 independent biological replicates, with *n* > 100 per replicate).

Niemann–Pick disease type C1 and C2 are caused by mutations in the *NPC1* and *NPC2* gene loci, leading to impaired intracellular cholesterol trafficking and subsequent accumulation of cholesterol and sphingolipids in lysosomes [4,12]. In *Drosophila*, the DIOPT recognized *Npc1a* and *Npc2a* as high confidence orthologs of human *NPC1* and *NPC2* genes, respectively (Table 2). Furthermore, structural alignment analysis yielded RMSD values below 1 Å for both the *NPC1*–*Npc1a* and *NPC2*–*Npc2a* protein product comparisons, thus indicating a high degree of structural similarity between the human and *Drosophila* proteins (during species evolution) (Figure 3A,E). Neuronal-specific downregulation of the *Npc1a* and *Npc2a* genes resulted in ~14% and ~58% reductions in mRNA expression levels (Figure 3B,F), respectively. Interestingly, albeit the moderate knockdown efficiency in the *Npc1a*-targeted flies, both male *Drosophila* Niemann–Pick models exhibited a significant decrease in lifespan along the brain–midgut axis with their median life expectancy being reduced by ~29 (Figure 3C; *Npc1α* targeting) and ~26 (Figure 3G; *Npc2α* targeting) days, following neuronal-specific downregulation, and ~20 (Figure 3D; *Npc1α* targeting) and ~17 (Figure 3H; *Npc2α* targeting) days, after midgut-specific targeting of the fly ortholog respective genes.

In contrast, female flies presented only a modest reduction in their lifespan, which could only be detected after ~50 days (post-eclosion), following neuronal-specific knockdown of either gene (Figure 3C,G), whereas their respective silencing specifically in the midgut tissues resulted in survival profiles similar to that of the controls (Figure 3D,H).

RNAi-mediated knockdown of the *Npc1a* gene in the nervous system caused an early and significant decline in the climbing ability of male flies. This deficit was statistically significant as early as day 10 post-eclosion and diverged sharply from the performance of control males, which maintained robust locomotor function (Figure S12A). Intriguingly, neuronal-specific silencing of the *Npc2a* gene in males did not affect their climbing performance relative to the controls (Figure S12B), indicating that *Npc2a* may play a secondary or redundant role compared to the *Npc1a* gene in locomotor function(s). In sharp contrast to the male phenotype, female flies of both genotypes exhibited climbing activities similar to the controls (Figure S12A,B). Strikingly, our findings indicate that even moderate reductions in *Npc1a* gene expression are sufficient to impair locomotor performance, occurring in a sex-specific manner during *Drosophila* aging.

#### 3.3.4. Tay–Sachs/Sandhoff Disease(s)

**Figure 4:**
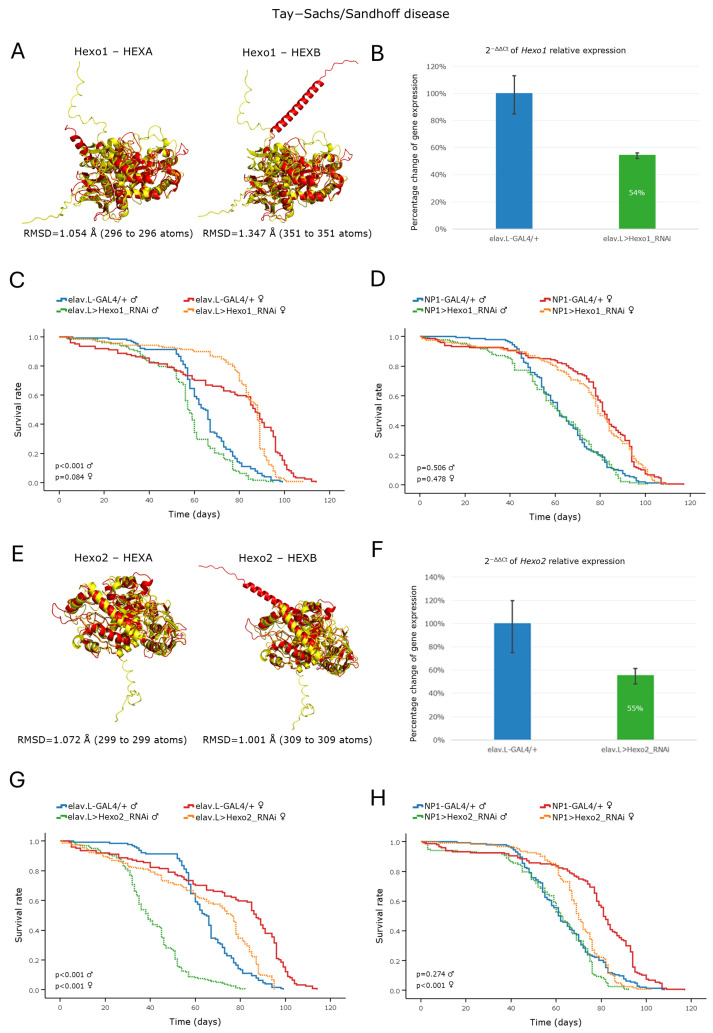
In vivo genetic modeling of Tay–Sachs and Sandhoff diseases in the *Drosophila* brain–midgut axis. (**A**–**D**) Functional analysis of *Hexo1* gene: (**A**) PyMOL-mediated structural alignment of the AlphaFold-predicted human *HEXA* and *HEXB* proteins (red) with their *Drosophila* ortholog protein being encoded by the *Hexo1* gene (yellow). (**B**) Relative mRNA expression levels of *Hexo1* in neuronal tissues (heads) of RNAi-targeted flies (elav > Hexo1_RNAi) compared to controls (elav-GAL4/+), assessed by RT-qPCR. (**C**) Kaplan–Meier survival curves of male and female flies, following neuronal-specific knockdown of *Hexo1*. (**D**) Lifespan profiles, after *Hexo1*-gene knockdown, specifically in midgut tissues (NP1 > Hexo1_RNAi), compared to control (NP1-GAL4/+). (**E**–**H**) Functional analysis of *Hexo2* gene: (**E**) Structural alignment of AlphaFold-predicted protein structures being derived from human *HEXA* and *HEXB* genes (red), and the *Drosophila* ortholog protein synthesized by the *Hexo2* gene (yellow). (**F**) Relative mRNA expression levels of *Hexo2* in neuronal tissues (elav.L > Hexo2_RNAi), versus control populations (elav.L-GAL4/+), assessed by RT-qPCR. (**G**) Survival curves of male and female flies being characterized by neuronal *Hexo2* knockdown, compared to control conditions. (**H**) Lifespan profiles, after *Hexo2*-gene silencing, specifically in the midgut tissues (NP1 > Hexo2_RNAi), compared to control (NP1-GAL4/+). Sample sizes: total *n* > 300 flies per genotype and sex (pooled from 3 independent biological replicates, with *n* > 100 per replicate).

The GM2 gangliosidoses are caused by defects in the degradation of GM2 ganglioside, leading to its accumulation primarily within neuronal cells. The degradation of GM2 ganglioside requires the lysosomal isoform Hex A, a β-Hexosaminidase enzyme composed of α- and β-subunits that are encoded by the *HEXA* and *HEXB* genes, respectively. Mutations in these genes result in the development of the Tay–Sachs (*HEXA*) and Sandhoff (*HEXB*) diseases [10,11]. A DIOPT analysis of the *Drosophila* genome identified three putative orthologs encoding β-N-Acetylhexosaminidase-like enzymes: the *Hexo1*, *Hexo2,* and *fdl* genes (Table 2). We focused on *Hexo1* and *Hexo2*, as they present the highest sequence homology and the most significant structural similarity to their human counterparts (Figure 4A,E).

RNAi-mediated knockdown of these genes in the nervous system caused a significant reduction in mRNA expression of ~46% for *Hexo1* (Figure 4B) and ~45% for *Hexo2* (Figure 4F) genes. Neuronal tissue-specific silencing of the *Hexo1* ortholog gene led to an age-dependent reduction in the median lifespan of male flies of ~8 days, whereas, in females, it caused a mild increase in early-life survival, followed by a decline in longevity during late(r)-life stages (Figure 4C). In contrast, neuronal silencing of *Hexo2* caused a comparatively more pronounced decrease in median lifespan of ~26 days for the male flies and of ~11 days for female populations (Figure 4G). Notably, RNAi-mediated downregulation of each gene in the midgut tissues proved to induce no significant impact on the lifespan profile of either gender (Figure 4D,H), thereby suggesting a limited, or redundant, functional role for the *Hexo1* and *Hexo2* genes in the *Drosophila* midgut environment during aging.

Taken together, the RNAi transgenic lines used in this study were found to be capable of recapitulating key pathological features of sphingolipidoses, thus reinforcing the exploitation of *Drosophila* as a reliable and powerful model organism for unmasking the molecular underpinnings of sphingolipidoses and related LSD pathologies during aging.

### 3.4. Modeling of Pompe Disease in Drosophila

#### Pompe Disease

**Figure 5:**
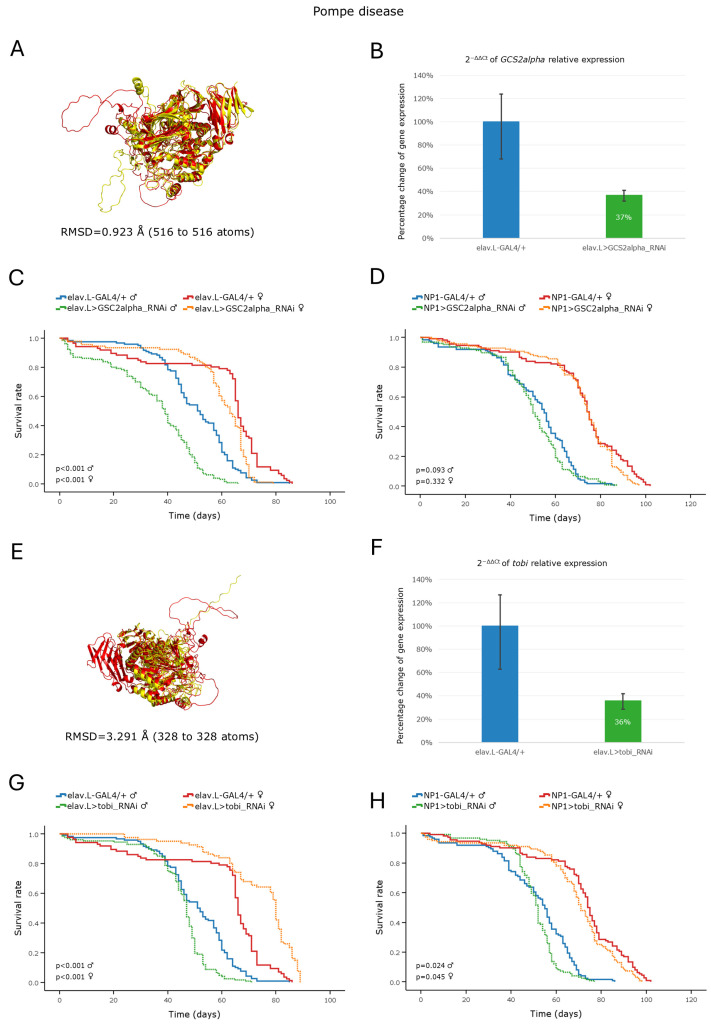
In vivo genetic modeling of Pompe disease in the *Drosophila* brain–midgut axis. (**A**–**D**) Functional analysis of the *GCS2alpha* gene: (**A**) PyMOL-mediated structural alignment of AlphaFold-derived protein products of the human *GAA* (red) and the *Drosophila* ortholog *GCS2alpha* (yellow) genes. (**B**) Relative mRNA expression levels of *GCS2alpha* in neuronal tissues (heads) of RNAi-targeted flies (elav > GCS2alpha_RNAi), compared to controls (elav-GAL4/+), assessed by RT-qPCR. (**C**) Survival curves of male and female flies following neuronal-specific knockdown of *GCS2alpha* versus control conditions. (**D**) Lifespan profiling after *GCS2alpha*-targeted downregulation, specifically in *Drosophila* midgut tissues (NP1 > GCS2alpha_RNAi), compared to control (NP1-GAL4/+). (**E**–**H**) Functional analysis of the *tobi* gene: (**E**) PyMOL-mediated structural alignment of AlphaFold-derived protein products of the human *GAA* (red) and the *Drosophila* ortholog *tobi* (yellow) genes. (**F**) Relative mRNA expression levels of *tobi* in RNAi-targeted neuronal tissues (elav.L > tobi_RNAi) versus control fly population (elav.L-GAL4/+), assessed by RT-qPCR. (**G**) Kaplan–Meier survival curves following neuronal-specific knockdown of *tobi* gene. (**H**) Survival profiles following the midgut-specific knockdown of the *tobi* gene (NP1 > tobi_RNAi), versus control (NP1-GAL4/+). Sample sizes: a total of *n* > 300 flies per genotype and sex (pooled from three independent biological replicates, with *n* > 100 per replicate).

Pompe disease is a glycogen storage disorder caused by a deficiency in Lysosomal acid-a-Glucosidase, which is encoded by the *GAA* gene and leads to intra-lysosomal glycogen accumulation [6]. In *Drosophila*, there is no single direct ortholog of the human *GAA* gene, hitherto pinpointed. However, three homolog genes can be, in silico, identified; *GCS2alpha*, which has a moderate DIOPT score and *tobi* and *CG33080*, which show low homology values (Table 2). For our study, we selected the *GCS2alpha* gene, as its cognate protein presents with the comparatively highest (predicted) homology and structural similarity to its human counterpart (Figure 5A). Furthermore, we also included the *tobi* gene (Figure 5E) due to its relatively higher DIOPT score compared to *CG33080,* respectively. The gene-silencing efficiency of our RNAi-based strategy, using a neuronal-specific driver, proved significantly strong for both of the ortholog genes, as the reduction in mRNA expression level was measured at ~63% for *GCS2alpha* (Figure 5B) and at ~64% for the *tobi* (Figure 5F) gene, versus control conditions. Neuronal-specific knockdown of *GCS2alpha* caused a significant decrease in the lifespan of male flies, a major pathology that was clearly detected from early adulthood (Figure 5C). Regarding *tobi*, its downregulation in males was linked to an age-dependent phenotype typified by reduced viability, observed after ~50 days from hatching (Figure 5G). Intriguingly, female flies did not suffer any negative effect on their lifespan after the silencing of either gene in the nervous system (Figure 5C,G). In fact, *tobi* knockdown was shown to improve, rather than deteriorate, female viability (Figure 5G). Our data strongly suggest for the a sexually dimorphic contribution of the *GCS2alpha* and *tobi* genes to the tight control of lifespan in *Drosophila*. Nevertheless, midgut-specific silencing of the *GCS2alpha* or *tobi* gene did not seem to cause any significant effect on life expectancy in either sex, strongly supporting their predominant functional involvement in the nervous, but not midgut, system during *Drosophila* aging (Figure 5D,H).

### 3.5. Modeling of Mucopolysaccharidoses in Drosophila

Mucopolysaccharidoses (MPSs) are caused by deficiencies in specific lysosomal hydrolases responsible for the sequential degradation of one or more glycosaminoglycans (GAGs), thus resulting in their lysosomal accumulation and ultimately in cellular dysfunction [8].

#### 3.5.1. Hurler Syndrome

**Figure 6:**
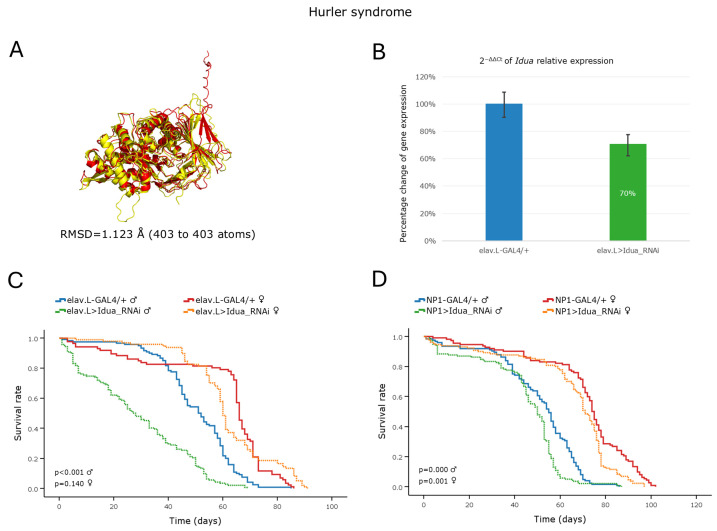
In vivo genetic modeling of Hurler syndrome in *Drosophila*, via the ortholog gene *Idua* targeting in the brain–midgut axis during aging. (**A**) PyMOL-mediated structural alignment of AlphaFold-derived protein structures that are encoded by the human *IDUA* (red) and its *Drosophila* ortholog *Idua* (yellow) genes. (**B**) Relative mRNA expression levels of *Idua* in neuronal tissues (heads) of RNAi-targeted flies (elav > Idua_RNAi) compared to the controls (elav-GAL4/+), assessed by RT-qPCR. (**C**) Survival curves of male and female transgenic flies following *Idua*-gene downregulation in the nervous system, compared to the controls. (**D**) Viability profiles, after midgut-specific silencing of the *Idua* gene (NP1 > Idua_RNAi), versus control conditions (NP1-GAL4/+). Sample sizes: a total of *n* > 300 flies per genotype and sex (pooled from three independent biological replicates, with *n* > 100 per replicate).

Hurler syndrome, or MPS type I, is caused by a deficiency in α-L-Iduronidase (encoded by the *IDUA* gene), leading to the pathological storage of dermatan- and heparan- sulfate inside the lysosomes of a wide range of tissues [13]. In *Drosophila*, a DIOPT analysis identified *Idua* as the single ortholog of the human *IDUA* gene, with a high-confidence homology score (Table 2) and an RMSD value of 1.123 Å (Figure 6A). Neuronal-specific RNAi-mediated knockdown of the *Idua* gene caused a ~30% reduction in gene expression (Figure 6B), which proved sufficient to induce a pathological phenotype in the male flies. These transgenic males exhibited a severely shortened lifespan (Figure 6C), with their median survival time being reduced by ~24 days and impaired climbing ability commencing as early as day one of their adult life (Figure S13). Strikingly, high mortality rates prevented the inclusion of 30-day-old male flies in the climbing assay, due to insufficient sample size. In contrast, female transgenic flies were characterized by absence of statistically significant changes in either life expectancy (Figure 6C) or climbing activity, apart from a slight improvement in locomotor performance observed at approximately day 30 (Figure S13). Of note is that midgut-specific knockdown of the *Idua* gene resulted in mildly reduced survival for both transgenic fly sexes compared to the controls (Figure 6D).

Altogether, the herein obtained results demonstrate that, following *Idua* suppression, *Drosophila* manifests key neurological and viability-related pathologies, which represent phenotypes indicative of human Hurler syndrome, thereby highlighting the strong potential of the “fly-*Idua*^RNAi^” genetic platform to serve as a reliable and effective in vivo animal disease model for mechanistically investigating Hurler syndrome pathogenesis and pre-clinically supporting high-throughput drug-screening systems, moving towards the discovery of novel therapeutic schemes and regimens.

#### 3.5.2. Hunter Syndrome

**Figure 7:**
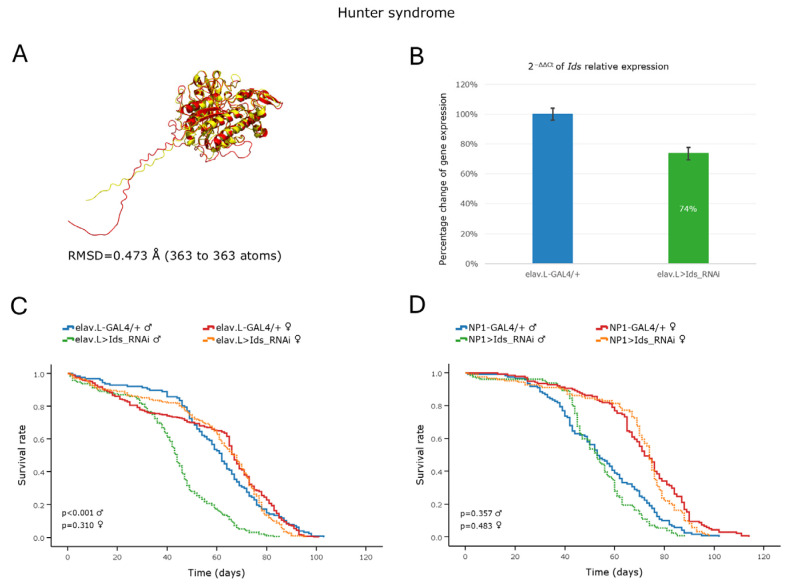
In vivo genetic modeling of Hunter syndrome in the *Drosophila* brain–midgut axis. (**A**) Structural alignment of the AlphaFold-predicted protein structures derived from the human *IDS* (red) and its *Drosophila* ortholog *Ids* (yellow) genes. Human proteins were aligned to the *Drosophila* structure using the PyMOL molecular graphics system. (**B**) Relative mRNA expression levels of *Ids* in neuronal tissues (heads) of RNAi-targeted flies (elav > Ids_RNAi) compared to controls (elav-GAL4/+), assessed by RT-qPCR. (**C**) Survival curves of transgenic flies from both sexes following *Ids* gene knockdown in the nervous system (elav.L > Ids_RNAi) versus control conditions (elav.L-GAL4/+). (**D**) Lifespan profiles of male and female flies after *Ids* gene silencing in midgut tissues (NP1 > Ids_RNAi) compared to control genetic crosses (NP1-GAL4/+). Sample sizes: a total of *n* > 300 flies per genotype and sex (pooled from three independent biological replicates, with *n* > 100 per replicate).

Hunter syndrome, or MPS type II, is a rare X-linked recessive disorder caused by a functional deficiency of the lysosomal enzyme Iduronate-2-sulfatase (encoded by the *IDS* gene in humans), which is critical for the catabolism of certain glycosaminoglycans (GAGs); the dermatan- and heparan-sulfate-GAG-species [14]. In *Drosophila*, a single ortholog gene, the *Ids*, can be identified in the 3rd chromosome, with a high DIOPT score (Table 2) and a strong structural similarity of its protein product to its human protein counterpart (an RMSD value as low as 0.473 Å; Figure 7A). Targeted, RNAi-mediated, knockdown of the *Ids* gene in neuronal cells led to a ~26% reduction in mRNA expression levels (Figure 7B). Despite this modest downregulation, male flies presented an age-dependent decrease in life expectancy, with their median survival time being reduced by ~18 days compared to control males (Figure 7C). In contrast, female flies were largely unaffected, producing survival curves comparable to those of the control populations (Figure 7C).

Similarly, tissue-specific knockdown of the *Ids* gene in *Drosophila* midgut tissues did not seem to significantly affect the lifespan profile of either fly sex setting (Figure 7D), further emphasizing the functional importance of the *Ids* gene product, specifically in the central nervous system (CNS). It may be that the remaining *Ids* activity, of ~74%, lies near the functional threshold of being capable of sufficiently maintaining viability in female, but not male, fly populations during aging.

#### 3.5.3. Sly Disease

**Figure 8:**
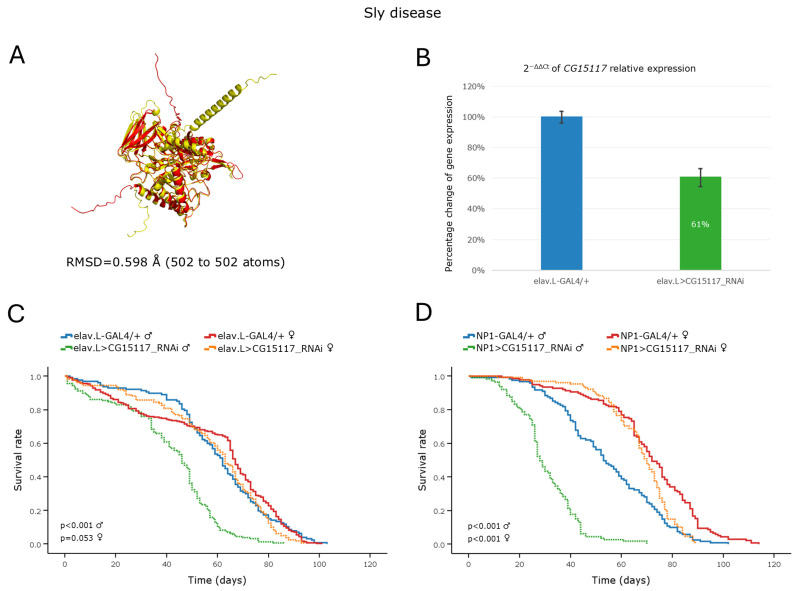
In vivo genetic modeling of Sly disease in *Drosophila*, via targeting of the *CG15117* ortholog gene in the brain–gut axis during aging. (**A**) PyMOL-mediated structural alignment of AlphaFold-derived protein structures encoded by the human *GUSB* (red) and its *Drosophila* ortholog *CG15117* (yellow) genes. (**B**) Relative mRNA expression levels of *CG15117* in neuronal tissues (heads) of RNAi-targeted flies (elav > CG15117_RNAi) compared to controls (elav-GAL4/+), assessed by RT-qPCR. (**C**) Lifespan curves of male and female transgenic flies, following nervous system-specific targeting of the *CG15117* gene (elav.L > CG15117_RNAi) compared to control conditions (elav.L-GAL4/+). (**D**) Survival profiles of transgenic flies (both sexes) after targeted *CG15117*-gene knockdown specifically within midgut tissues (NP1 > CG15117_RNAi) compared to control genetic crosses (NP1-GAL4/+). Sample sizes: a total of *n* > 300 flies per genotype and sex (pooled from three independent biological replicates, with *n* > 100 per replicate).

Sly disease, or MPS type VII, is an autosomal recessive LSD caused by mutations in the human *GUSB* gene, which encodes the β-Glucuronidase enzyme. Loss of this enzyme leads to the accumulation of undegraded, or partially degraded, glycosaminoglycans (GAGs), ultimately resulting in widespread cellular dysfunction [14]. In *Drosophila*, 3 orthologs of the human *GUSB* gene have been identified. Among them, *CG15117* exhibits the highest DIOPT score (Table 2) and a notably low RMSD value of 0.598 Å (Figure 8A), indicative of their strong structural similarity. In the herein developed *Drosophila* model of Sly disease, RNAi-mediated knockdown of the *CG15117* gene, specifically within the nervous system, revealed a modest reduction of ~39% in mRNA expression levels (Figure 8B). Male transgenic flies with neuronal-specific downregulation of the *CG15117* gene were characterized by an age-dependent decline in lifespan, with a median reduction of ~16 days, compared to control males (Figure 8C). In contrast, female transgenic flies provided similar-to-control survival curves under the same growth conditions (Figure 8C).

Interestingly, midgut-specific knockdown of the *CG15117* gene also caused a pronounced, sex-dependent, lifespan impairment pattern. Male transgenic flies presented a significantly shortened lifespan, as clearly indicated by their reduced median and maximum lifespan of ~25 and ~40 days, respectively (Figure 8D). Female transgenic flies were comparatively less affected, with only a slight decrease in maximum lifespan being observed, thereby suggesting a male-specific vulnerability to *CG15117* loss in both neuronal and midgut tissues. Negative-geotaxis assay of RNAi-mediated, *CG15117*-targeted flies, specifically within the nervous system, unveiled a progressive, age-dependent decline in locomotor activity for both sexes (Figure S14). Although *CG15117*-downregulated flies presented near to normal climbing activity during the initial days of their adult life, with only a mild early decline being observed in females, their motor performance deteriorated significantly from day 10 onward. By this stage, their climbing efficiency closely resembled that of 20-day-old control flies, thereby indicating an accelerated onset of age-dependent locomotor impairment (Figure S14).

Taken together, the majority of the herein developed *Drosophila* models of Mucopolysaccharidoses (MPSs) successfully recapitulate key pathological features of MPS disorders, such as a shortened lifespan and progressive motor decline. These invertebrate models provide a powerful platform for conducting genetic screens in vivo in order to mechanistically illuminate MPSs, identify genetic modifiers, and conduct rapid, reliable, comprehensive, and cost-effective drug-screening trials that are unfeasible to implement in typical vertebrate (e.g., zebrafish and mouse) model organisms.

## 4. Discussion

Most lysosomal storage disorders (LSDs) lack effective treatments, rendering genome editing one of the most promising therapeutic strategies. However, before these genome editing tools can be applied in humans, several critical steps must precede their application, ranging from in vitro testing to clinical trials. To maximize safety and gather extensive preliminary data, in vivo modeling using invertebrates has gained major attention in recent years [15,17]. These organisms offer a wide array of genetic tools and allow the in vivo study of various biological pathways and therapeutic approaches, in less time and with fewer ethical concerns than those in mammals. *Drosophila melanogaster* is a well-established invertebrate model system that offers an ideal background for genetic and biological studies of different human pathologies, as it contains functional orthologs for ~75% of human disease-related genes [41]. *Drosophila* also features a plethora of genetic tools, including the GAL4/UAS, CRISPR/Cas9, and RNAi molecular platforms, which allow cell/tissue-specific gene targeting/downregulation [42,43]. Importantly, *Drosophila* models have been successfully utilized as robust in vivo screening platforms during the early stages of drug discovery. Numerous studies demonstrate their effectiveness for both primary high-throughput screening and secondary validation of biologically active compounds across a wide range of human diseases, ranging from neurodegeneration to cancer [44,45]. Of particular relevance to this work, the core cell biology of the lysosomal–autophagic system is highly conserved between flies and mammals. This conservation supports the translational validity of using *Drosophila* LSD models to identify pharmacological targets and pathways that emulate the pathological mechanisms observed in human patients.

In the present study, we utilized the binary GAL4/UAS and RNAi genetic systems, to selectively knockdown fly orthologs of human LSD-related genes within the brain–midgut axis during aging. Employment of commercially available transgenic strains, directly obtained from the Bloomington *Drosophila* Stock Center (BDSC; Bloomington, IN, USA), enabled us to systemically investigate their pathological phenotypes in vivo. These findings provide a powerful invertebrate model for future studies, to broadly explore and elucidate the molecular mechanisms controlling LSD pathology (initiation and progression) and to facilitate the development of novel genetic and pharmacological therapeutic strategies. Finally, it is important to note that the knockdown efficiencies reported here are likely underestimated. Because qRT-PCR was performed on whole adult heads, the inclusion of non-neuronal tissues, where the elav driver is inactive, dilutes the measurable effect of RNAi-mediated silencing within neuronal cells.

Sphingolipidoses represent a sub-category of LSDs that are developed by deficiencies in the enzymes responsible for the catabolism of sphingolipids and they mainly affect nervous-system and peripheral-organ tissues. Gaucher disease (GD) is the most prevalent form and derives from deficiencies in the β-Glucocerebrosidase (GBA1) enzyme, leading to toxic accumulation of glucosylceramide [10]. Utilization of mouse models for GD has proven challenging and limited, due to the elevated perinatal lethality associated with *GBA1* gene mutations [10,46].

Hence, working towards the establishment of a new in vivo model for the disease (GD), we investigated the impact of downregulating the *Drosophila Gba1a* and *Gba1b* orthologs of the human *GBA1* gene within the brain–midgut axis during aging. Our results revealed a marked reduction in lifespan and climbing ability, with male flies being more severely affected, compared to female populations. A previous study of *Drosophila* Minos-insertion mutants of the *GBA1* orthologs reported that the *Gba1b* mutants exhibited a shortened lifespan and an impaired climbing ability, whereas *Gba1a* mutants did not present significant pathologies [47]. Although the *Drosophila Gba1a* and *Gba1b* fly orthologs show differential tissue-expression patterns, with *Gba1a* being primarily expressed in the midgut and *Gba1b* being detected in the adult head and fat body [48], both genes seem to affect fly longevity and kinetic ability in a similar pattern when downregulated in brain and midgut tissues.

Therefore, our findings demonstrate that both *Gba1a* and *Gba1b* are essential for motor performance and survival in *Drosophila*. The progressive loss of locomotor activity observed herein likely mirrors the neuronal cell loss and resulting neurotoxicity characteristic of human GD [10]. The distinct sexual dimorphism of lifespan and climbing phenotypes observed in our *Drosophila* GD models align with a growing body of evidence linking sexual dimorphism to fundamental variations in metabolic homeostasis, stress responses, hormonal regulation, immune responses, and reproductive physiology [36,37,38,39,49]. In *Drosophila*, female physiology is heavily geared towards lipid accumulation and protein synthesis to support egg production, whereas male metabolism is optimized for carbohydrate utilization and locomotor activity [39,49]. This intrinsic dimorphism likely explains the sex-specific phenotypes observed in our GD RNAi models. Since *Gba1* and *Gba1b* are critical for glucosylceramide catabolism, their depletion would differentially impact the high-demand lipid environment of the female gut compared to the male gut, reflecting the divergent aging trajectories of the two sexes. Taken together, our findings strongly support *Drosophila* as a powerful and versatile in vivo model for GD, providing insights into its genetic and pathophysiological mechanisms, including sex-specific disease manifestations.

Fabry is an X-linked recessive sphingolipidosis caused by a deficiency in the lysosomal enzyme α-Galactosidase A due to mutations in the human *GLA* gene [7]. Interestingly, in our model system herein investigated, genetic downregulation of the fly *GLA* ortholog *CG5731* proved capable of more severely affecting female flies, in terms of both life expectancy and climbing capacity, compared to male populations. Of note is that the sex-linked inheritance pattern that has been observed in humans cannot be directly applied in *Drosophila*, since male flies upregulate their single X chromosome via dosage compensation [50]; additionally, *CG5731* is not an X-linked gene. A mechanistic explanation for the comparatively increased sensitivity detected in female flies may be associated with sex- and/or tissue-specific gene-expression programs and differences in metabolic/nutritional demands and/or hormonal pathway/network activities. Moderate homologies might also reflect redundant or compensatory functions by other enzymes or alternative mechanisms in *Drosophila* that are absent or less efficient in humans. In a mouse model of the disease (FD), both male and female mice deficient in α-Gal A manifested a clinically normal phenotype at the 10th–14th weeks of age, thus rendering FD modeling in this mammalian system, both challenging and limited [51]. However, in a study of *Drosophila* transgenic populations expressing the human mutant *GLA* (variant) forms A156V and A285D, significant locomotor dysfunction and reduced lifespan were observed compared to control flies (expressing the human wild-type enzyme). Strikingly, these phenotypes could be ameliorated with Migalastat (FD medication) treatment [52].

Altogether, our RNAi-based genetic platform, which targets the endogenous expression of the *CG5731* fly gene (a human *GLA* homolog) specifically within the brain–midgut axis during aging, may offer a powerful, reliable, multifaceted, dynamic, and sensitive in vivo model system for comprehensively studying FD, to enable efficient drug screening and to illuminate underlying disease mechanisms.

Niemann–Pick type C disease (NPC) is a neurodegenerative disorder classified into types C1 and C2 depending on the respective human gene (*NPC1* or *NPC2*) that is mutated. It is characterized by abnormalities in the intracellular transport of endocytosed cholesterol, which leads to the accumulation of cholesterol and sphingolipids within endo-lysosomes [6,12]. In the present study, we investigated the consequences of RNAi-mediated knockdown of the *Drosophila* orthologs *Npc1a* and *Npc2a* within the brain–midgut axis during aging. The obtained male transgenic flies were characterized by a reduced lifespan and locomotor dysfunction with organ-specific (brain or midgut) targeting, in contrast to the female flies, which exhibited near-normal phenotypes. This pronounced male-specific vulnerability highlights a significant sexual dimorphism in the susceptibility to defects in cholesterol trafficking. In *Drosophila*, females possess specialized metabolic adaptations to buffer lipid fluctuations to support oogenesis [39,49], likely conferring a degree of resilience against the intracellular cholesterol accumulation associated with NPC pathology. Conversely, since lysosomal lipid accumulation is known to trigger oxidative stress and mitochondrial dysfunction, the male-specific lethality observed herein likely reflects a lower physiological threshold for lipotoxicity in males, rendering them disproportionately vulnerable to the loss of *Npc1a* or *Npc2a* functions [53].

A previous study in *Drosophila*, using loss of function mutants of the *Npc1a* gene, revealed developmental arrest at the first larval stage [54], thus rendering age-dependent pathologies during adulthood impossible to profiled. Strikingly, in our model, although the viability patterns for the two genes are largely similar, the relative expression of the *Npc1a* gene was less markedly reduced compared to the *Npc2a* respective one (Figure 3B,F). This indicates that even a modest decrease in the *Npc1a* gene-expression level within the nervous system setting is sufficient to trigger a pathological phenotype, thereby highlighting the essential role(s) of *Npc1a* in *Drosophila* well-being during aging.

In toto, our genetic approach provides a powerful, trustworthy, and manageable model system for mechanistically illuminating and therapeutically advancing Niemann–Pick type C disease (NPC) in vivo.

GM2 gangliosidoses are characterized by excessive accumulation of ganglioside GM2 species and related glycolipids in the lysosomes. The main forms include Tay–Sachs disease (TSD), caused by mutations in the *HEXA* gene, and Sandhoff disease (SD), caused by mutations in the *HEXB* gene [9,10,11]. In *Drosophila*, three genes (*Hexo1*, *Hexo2,* and *fdl*) have been identified as encoding β-Hexosaminidase-like enzymes based on sequence homologies to human Hexosaminidases [55,56]. Strikingly, RNAi-mediated downregulation of the *Hexo2* (but not *Hexo1*) gene, specifically within the brain, revealed a remarkable reduction in the life expectancy of *Drosophila* transgenic male flies (Figure 4G). Given that GM2 gangliosidoses are known to predominantly affect the central nervous system (CNS) [10,11], our results point to the essential contribution of certain β-Hexosaminidases to neuronal development and CNS/brain functionality in *Drosophila* during aging, thereby validating the model’s relevance to molecularly investigating GM2 gangliosidoses-induced neuro-pathologies in vivo.

Mucopolysaccharidoses (MPSs) comprise a class of 11 lysosomal storage disorders, with each one being derived from driver deficiency in the activity of a distinct lysosomal hydrolase; they all belong to a family of enzymes that are critically involved in the sequential degradation of glycosaminoglycans (GAGs). MPS I and MPS II sub-types were typically classified among the first syndromes identified within this group [8]. In our Hurler syndrome (HLS) (MPS I; α-L-Iduronidase deficiency) in vivo model, although the expression of the *IDUA* gene was not severely downregulated within the brain–midgut axis, reduced lifespan and locomotor deficiency were observed. Of note is that a distinct study method regarding HLS modeling using a similar strategy, but different RNAi strains, which can still target the same *Drosophila IDUA* ortholog (*CG6201*) gene, has been previously reported by Filippis et al. [57]. However, in their set of experiments, although flies with reduced expression of the *IDUA* gene in neuronal and glial cells presented locomotion deficiencies, they, unexpectedly, manifested a longer lifespan compared to the controls [57].

Hence, our *Drosophila* HLS model represents an invaluable, powerful, informative, constructive, manageable, and novel complement to the existing biological tool for genetically dissecting disease mechanisms and systemically expanding the repertoire of experimental in vivo models hitherto available for deeper investigation of HLS pathology, both mechanistically and therapeutically, for the maximum benefit of humans.

Hunter syndrome (HNS) (MPS II; Iduronate-2-sulphatase deficiency) is an X-linked recessive LSD. Remarkably, in our invertebrate model system, only males exhibited a notable reduction in life expectancy, a pathological phenotype that is genetically associated with the sex-dependent nature of HNS in humans. The genetic modeling of HNS in *Drosophila* has been previously described using the same RNAi strains, with the authors concluding that residual *Ids* activity may be sufficient to rescue MPS II-related pathologies, since, in their lethality assays, the survival from larva to pupa and the metamorphosis to the adult phase were not affected [58]. In contrast to their argument that the engagement of RNAi-dependent transgenic technology for MPS II knockdown is not an effective strategy, our data strongly suggest that, under certain circumstances and specific settings, the exploitation of male flies as a novel and reliable model system for HNS-pathology research in vivo should not be ignored or disregarded.

Employment of our Sly disease (SLD) (MPS VII; β-Glucuronidase deficiency) model demonstrated a remarkable reduction in the viability of male flies along the brain–midgut axis, together with a progressive decline in locomotor activity for both sexes during aging. A *Drosophila* model of MPS VII, developed by knocking out the *CG2135* gene, the fly ortholog of human *GUSB*, has been previously established by Bar et al., successfully recapitulating key features of SLD such as shortened lifespan, motor deficiencies, and neurological abnormalities [59]. Notably, *Drosophila* possesses two orthologs of the human *GUSB* gene; the *CG2135* (*βGlu*) and the *CG15117* gene, with the latter exhibiting a slightly higher similarity score in the DIOPT analysis [20]. Although Bar et al. found that CG15117 was 6-fold less active than CG2135, our results clearly demonstrate that targeted downregulation of *CG15117*, in either brain or midgut tissues, critically compromises male fly viability during aging, thereby strongly suggesting its (*CG15117*) beneficial utilization as an additional, but important and powerful, screening tool for SLD research in vivo.

Altogether, we have herein identified the *Drosophila* orthologs of the genes responsible for the most common LSDs in humans and systematically screened them for “patho-phenotypic” effects on life expectancy and climbing proficiency, specifically within the brain–midgut axis during aging, suitably engaging the GAL4/UAS binary transgenic system, in combination with the RNAi-mediated gene-silencing platform. Most of these in vivo LSD models in *Drosophila* proved capable of successfully recapitulating the key disease phenotypes that have been identified in humans, including significantly reduced lifespan and progressive climbing deficiency, which serve as a proxy for neuro-muscular disintegration, in an age- and sex-dependent manner (Table 3). However, we acknowledge the inherent limitations of this approach, including the variable knockdown efficiency of RNAi compared to null mutants and the tissue-restricted nature of the GAL4 drivers, which precludes the assessment of systemic contributions from non-neuronal or non-midgut tissues.

Notwithstanding these constraints, the consistent phenotypic parallels undoubtedly underline the value and importance of *Drosophila* as a robust, reliable, powerful, rapid, multifaceted, versatile, and manageable invertebrate model system. *Drosophila* LSD models are ideal for high-throughput genetic and pharmacological in vivo screenings, which aim for pathological-phenotype rescues, while also providing invaluable insights into the underlying molecular and neurological mechanisms that tightly control LSD-specific pathologies.

## 5. Conclusions

We present a comprehensive, DIOPT-derived resource cataloging nearly all of the human LSD-related genes and their *Drosophila* orthologs, complemented by available RNAi reagents from major stock centers. Structural analysis via AlphaFold confirms the high degree of evolutionary conservation between human and *Drosophila* species, validating the molecular relevance of the fly model. Through tissue-specific targeting of LSD-related genes in the brain and midgut, we successfully recapitulated key LSD pathologies, thus demonstrating the robustness of these models during aging. Notably, our systematic screening reveals a marked sexual dimorphism in disease susceptibility (summarized in Table 3), likely driven by the distinct metabolic profiles of male and female physiology. Collectively, this phenotypic “atlas” establishes a versatile in vivo platform for dissecting the molecular mechanisms of LSDs and accelerating high-throughput therapeutic discoveries.

## Figures and Tables

**Table 1:** A list of *Drosophila* RNAi strains and the specific genotypes utilized in this study.

Drosophila Ortholog	Stock ID (RRID)	Genotype
*Gba1a*	39064	y [1] sc[*] v [1] sev [21]; P{y[+t7.7] v[+t1.8] = TRiP.HMS01984}attP2
*Gba1b*	38977	y [1] sc[*] v [1] sev [21]; P{y[+t7.7] v[+t1.8] = TRiP.HMS01893}attP40
*CG7997*	63655	y [1] v [1]; P{y[+t7.7] v[+t1.8] = TRiP.HMJ30222}attP40
*CG5731*	67025	y [1] sc[*] v [1] sev [21]; P{y[+t7.7] v[+t1.8] = TRiP.HMS05491}attP40
*Npc1a*	37504	y [1] sc[*] v [1] sev [21]; P{y[+t7.7] v[+t1.8] = TRiP.HMS01646}attP40
*Npc2a*	38237	y [1] sc[*] v [1] sev [21]; P{y[+t7.7] v[+t1.8] = TRiP.HMS01681}attP40
*Hexo1*	67312	y [1] sc[*] v [1] sev [21]; P{y[+t7.7] v[+t1.8] = TRiP.HMC06416}attP40
*Hexo2*	57199	y [1] sc[*] v [1] sev [21]; P{y[+t7.7] v[+t1.8] = TRiP.HMC04581}attP40
*GCS2alpha*	34334	y [1] sc[*] v [1] sev [21]; P{y[+t7.7] v[+t1.8] = TRiP.HMS01322}attP2
*tobi*	53379	y [1] v [1]; P{y[+t7.7] v[+t1.8] = TRiP.HMJ02101}attP40
*Idua*	64931	y [1] sc[*] v [1] sev [21]; P{y[+t7.7] v[+t1.8] = TRiP.HMC05804}attP2
*Ids*	51901	y [1] v [1]; P{y[+t7.7] v[+t1.8] = TRiP.HMC03475}attP40
*CG15117*	33693	y [1] sc[*] v [1] sev [21]; P{y[+t7.7] v[+t1.8] = TRiP.HMS00562}attP2

**Table 2:** *Drosophila* orthologs of human Lysosomal Storage Disorder (LSD)–associated genes identified via DIOPT.

Lysosomal Storage Disorders (*LSDs*)	Human Gene	Protein Name	*Drosophila* Ortholog	Homology (*Rank*)/ (*DIOPT Score*)	RNAi Strain
**1**. **Autosomal recessive spastic** **paraplegia type 48 (SPG48)**	*AP5Z1*	Adaptor-related protein complex 5 subunit zeta 1	*Lpin*/*CG8709*	Low (1)	63614 ^1^
					77170 ^1^
**2**. **Disorders of lysosomal amino acid transport**					
A. Cystinosis	*CTNS*	Cystinosin, Lysosomal Cystine transporter	*Ctns*/ *CG17119*	High (16)	40823 ^1^
B. Free sialic acid storage disease (free SASD)					
(a) Salla disease (SD)	*SLC17A5*	Sialin, Solute carrier family 17 member 5	*VGlut2*/*MFS9*/*CG4288*	High (10)	29305 ^1^
(b) Intermediate severity Salla disease					v104145 ^2^
(c) Infatile free sialic acid storage disease (ISSD)					
**3. Disorders of sialic acid metabolism**					
Sialuria	*GNE*	Glucosamine (UDP-N-acetyl)-2-epimerase/N-Acetyl-mannosamine kinase	-	-	-
**4. Glycoproteinoses**					
A. Mucolipidoses (ML)					
(a) ML type II α/β: Inclusion (I)- cell disease	*GNPTAB*	N-Acetyl-glucosamine-1-phosphotransferase subunits α/β	*Gnptab*/ *CG8027*	High (15)	v109400 ^2^
(b) ML type III: Pseudo-Hurler polydystrophy:					
type III α/β	*GNPTG*	N-Acetyl-glucosamine-1-phosphotransferase subunit γ	*GCS2beta*/ *CG6453*	Moderate (3)	35008 ^1^
type III γ			*CG7685*	Low (2)	62254 ^1^
(c) ML type IV: Sialolipidosis	*MCOLN1*	Mucolipin 1, Mucolipin transient receptor potencial (TRP) cation channel 1	*CG42638*	Moderate (14)	44098 ^1^
			*Trpml*/ *CG8743*	Moderate (14)	31294 ^1^
					31673 ^1^
					v108088 ^2^
					v45989 ^2^
B. Oligosaccharidoses					
(a) α-Mannosidosis	*MAN2B1*	Lysosomal α-Mannosidase, Mannosidase alpha class 2B member 1	*LManII*/ *CG6206*	High (16)	53294 ^1^
			*LManI*/ *CG5322*	Moderate (14)	44473 ^1^
			*LManV*/ *CG9466*	Moderate (14)	v104300 ^2^
					v13040 ^2^
			*LManIV*/ *CG9465*	Moderate (14)	66992 ^1^
			*LManIII*/ *CG9463*	Moderate (13)	v15589 ^2^
					v48063 ^2^
			*LManVI*/ *CG9468*	Moderate (12)	61216 ^1^
			*alpha*-*Man-IIa*/*CG18802*	Low (3)	v5838 ^2^
			*alpha-Man-IIb*/*CG4606*	Low (2)	v108043 ^2^
					v42652 ^2^
(b) β-Mannosidosis	*MANBA*	β-Mannosidase	*beta-Man*/ *CG12582*	High (14)	53272 ^1^
(c) Fucosidosis	*FUCA1*	α-L-Fucosidase 1	*Fuca*/ *CG6128*	High (13)	-
(d) Aspartyglucosaminuria (AGU)	*AGA*	Aspartylglucosaminidase	*CG1827*	High (14)	65141 ^1^
			*CG10474*	High (14)	51444 ^1^
			*CG4372*	Moderate (8)	v36431 ^2^
			*CG7860*	Low (2)	v108281 ^2^
					v34394 ^2^
			*Tasp1*/ *CG5241*	Low (2)	64907 ^1^
(e) α-Ν-Acetyl- galactosaminidase deficiency (NAGA deficiency): Schindler disease:					
type I: Infantile onset Neuroaxonal dystrophy	*NAGA*	α-N-Acetyl-galactosaminidase	*CG5731*	High (16)	67025 ^1^
type II: Kanzaki disease			*CG7997*	Moderate (15)	63655 ^1^
type III: Intermediate severity					57781 ^1^
(f) Galactosialidosis: Goldberg syndrome	*CTSA*	Protective protein Cathepsin A, and a secondary deficiency in β-Galactosidase and Neuraminidase-1	*CG4572*	Moderate (4)	34337 ^1^
			*CG32483*	Low (2)	v106263 ^2^
					v22976 ^2^
			*hiro*/*CG3344*	Low (2)	v110402 ^2^
					v15213 ^2^
			*CG31821*	Low (2)	v106059 ^2^
					v15496 ^2^
			*CG31823*	Low (2)	66941 ^1^
					67027 ^1^
(g) Sialidosis:					
type I (ST-1): Cherry-red spot- myoclonus syndrome	*NEU1*	Neuraminidase-1, Lysosomal Sialidase	-	-	-
type II (ST-2): Mucolipidosis I					
**5. Lysosomal acid phosphatase** **deficiency**					
**6. Glycogen storage disease(s)** **[GSD(s)]**					
GSD type II (due to acid maltase deficiency): Pompe disease	*GAA*	Lysosomal α-Glucosidase, Acid maltase	*GCS2alpha*/ *CG14476*	Moderate (5)	34334 ^1^
			*tobi*/ *CG11909*	Low (3)	53379 ^1^
			*CG33080*	Low (2)	42554 ^1^
GSD due to LAMP-2 deficiency: Danon disease	*LAMP2*	Lysosomal-associated membrane protein 2	*Lamp1*/ *CG3305*	Moderate (8)	38335 ^1^
					38254 ^1^
			*CG32225*	Low (3)	v102345 ^2^
					v5383 ^2^
**7. Mucopolysaccharidoses (MPSs)**					
MPS I					
Hurler syndrome (MPSIH)	*IDUA*	α-L-Iduronidase	*Idua*/*CG6201*	High (14)	64931 ^1^
Hurler-Scheie syndrome (MPSIH/S)					
Scheie syndrome (MPSIS)					
MPS II: Hunter syndrome					
type A (MPSIIA), severe form	*IDS*	Iduronate 2-sulfatase	*Ids*/*CG12014*	High (18)	51901 ^1^
type B (MPSIIB), attenuated form					63004 ^1^
MPS III: Sanfilippo syndrome					
type A (MPSIIIA)	*SGSH*	N-Sulfoglucosamine sulfohydrolase	*Sgsh*/ *CG14291*	High (16)	v107384 ^2^
					v16897 ^2^
type B (MPSIIIB)	*NAGLU*	N-Acetyl-α-glucosaminidase	*Naglu*/ *CG13397*	High (17)	51808 ^1^
type C (MPSIIIC)	*HGSNAT*	Heparan-α-glucosaminide N-acetyltransferase	*Hgsnat*/ *CG6903*	High (15)	33423 ^1^
type D (MPSIIID)	*GNS*	N-Acetylglucosamine-6-sulfatase	*Gns/* *CG18278*	High (15)	28520 ^1^
					51878 ^1^
					v109944 ^2^
					v22936 ^2^
MPS IV: Morquio syndrome					
type A (MPSIVA)	*GALNS*	N-Acetylgalactosamine-6-sulfatase	*CG7408*	Moderate (3)	65359 ^1^
			*Gns*/ *CG18278*	Moderate (3)	28520 ^1^
					51878 ^1^
			*CG7402*	Moderate (3)	v103947 ^2^
					v37302 ^2^
			*CG32191*	Moderate (3)	v101578 ^2^
					v14294 ^2^
type B (MPSIVB)	*GLB1*	β-Galactosidase 1	*Ect3*/*CG3132*	Moderate (15)	62217 ^1^
			*Gal*/*CG9092*	Moderate (14)	42922 ^1^
					50680 ^1^
MPS VI: Maroteaux–Lamy syndrome	*ARSB*	Arylsulfatase B	*CG7402*	High (13)	v103947 ^2^
					v37302 ^2^
MPS VII: Sly disease	*GUSB*	β-Glucuronidase	*CG15117*	High (17)	33693 ^1^
			*beta-Glu*/*CG2135*	Moderate (15)	62236 ^1^
			*beta-Man*/ *CG12582*	Low (2)	53272 ^1^
MPS IX: Hyaluronidase deficiency	*HYAL1*	Hyaluronidase 1	-	-	-
**8. Neuronal ceroid lipofuscinoses** **(NCL): Batten disease**					
CLN1: Haltia–Santavuori disease /Hagberg–Santavuori disease/ Santavuori disease (INCL)	*PPT1*	Palmitoyl-protein thioesterase 1	*Ppt1*/ *CG12108*	High (14)	55331 ^1^
					62291 ^1^
					25952 ^1^
			*Ppt2*/ *CG4851*	Low (3)	28362 ^1^
					v106819 ^2^
					v1459^2^
CLN2: Jansky–Bielschowsky disease (LINCL)	*TPP1*	Tripeptidyl peptidase 1	-	-	-
CLN3: Batten–Spielmeyer–Sjogren disease (JNCL)	*CLN3*	Battenin, Endosomal transmembrane protein	*Cln3*/ *CG5582*	High (14)	35734 ^1^
CLN4: Parry disease/Kufs disease type A and B (ANCL)	*DNAJC5*	Cysteine string protein, DnaJ Heat shock protein family (Hsp40) member C5	*Csp*/*CG6395*	High (14)	33645 ^1^
					31290 ^1^
					31669 ^1^
			*CG7130*	Low (2)	57854 ^1^
			*CG7133*	Low (2)	60459 ^1^
					42820 ^1^
			*l(3)80Fg*/ *CG40178*	Low (2)	44578 ^1^
CLN5: Finnish variant	*CLN5*	Ceroid-lipofuscinosis neuronal protein 5	-	-	-
CLN6: Lake–Cavanagh or Indian variant	*CLN6*	Transmembrane ER protein	-	-	-
CLN7: Turkish variant	*MFSD8*	Major-facilitator superfamily domain containing 8	*Cln7*/ *CG8596*	High (16)	61960 ^1^
					55664 ^1^
			*rtet*/*CG5760*	Low (2)	v110473 ^2^
					v44002 ^2^
CLN8: Northern epilepsy/ Epilepsy mental retardation	*CLN8*	Protein CLN8, Transmembrane ER and ERGIC protein	*CG17841*	Moderate (3)	34948 ^1^
CLN9	N/A	N/A			
CLN10: Congenital NCL	*CTSD*	Cathepsin D, Lysosomal Aspartyl peptidase/protease	*cathD*/ *CG1548*	High (16)	28978 ^1^
					53882 ^1^
					55178 ^1^
CLN11	*GRN*	Granulin (precursor)	*CG15011*	Low (1)	58284 ^1^
					31589 ^1^
			*NimC2*/ *CG18146*	Low (1)	25960 ^1^
					v3120 ^2^
					v36261^2^
CLN12: Kufor–Raked syndrome/ PARK9/Juvenile Parkinsonism—NCL	*ATP13A2*	Cation-transporting ATPase 13A2, PARK9	*anne*/ *CG32000*	Moderate (13)	44005 ^1^
					30499 ^1^
			*CG6230*	Low (3)	77371 ^1^
			*SPoCk*/ *CG32451*	Low (2)	44040 ^1^
					28352 ^1^
CLN13	*CTSF*	Cathepsin F	*CtsF*/ *CG12163*	High (14)	33955 ^1^
CLN14: Progressive myoclonic epilepsy type 3	*KCTD7*	Potassium channel tetramerization domain containing 7	*Ktl*/*CG10830*	Moderate (2)	57171 ^1^
					25848 ^1^
			*CG14647*	Moderate (2)	60064 ^1^
					27032 ^1^
			*twz*/ *CG10440*	Moderate (2)	57397 ^1^
					25846 ^1^
**9. Pycnodysostosis: Toulouse-Lautrec** **syndrome—Osteopetrosis** **acro-osteolytica**	*CTSK*	Cathepsin K	*CtsL1*/ *CG6692*	Moderate (8)	41939 ^1^
					32932 ^1^
**10. Sphingolipidoses**					
A. Acid sphingomyelinase deficiency (ASMD)					
Niemann–Pick disease types A and B	*SMPD1*	Sphingomyelin phosphodiesterase	*Asm*/*CG3376*	High (17)	36760 ^1^
			*CG15533*	Moderate (8)	36761 ^1^
			*CG15534*	Moderate (8)	36762 ^1^
			*CG32052*	Moderate (6)	36763 ^1^
B. Autosomal recessive cerebellar ataxia with late-onset spasticity (due to GBA2 deficiency)	*GBA2*	β-Glucosylceramidase 2	*CG33090*	High (18)	36688 ^1^
C. Encephalopathy due to prosaposin deficiency— Combined PSAP deficiency (PSAPD)	*PSAP*	Prosaposin	*Sap-r*/ *CG12070*	High (14)	v51129 ^2^
					v51130 ^2^
D. Fabry disease—Angiokeratoma corporis diffusum	*GLA*	α-Galactosidase A	*CG7997*	Moderate (14)	63655 ^1^
					57781 ^1^
			*CG5731*	Moderate (13)	67025 ^1^
E. Farber lipogranulomatosis	*ASAH1*	Acid Ceramidase	-	-	-
F. Gangliosidoses					
(a) GM1 gangliosidosis: Landing disease:					
type I (infantile): Norman–Landing disease	*GLB1*	β-Galactosidase	*Ect3*/*CG3132*	Moderate (15)	62217 ^1^
type II (juvenile—late infantile)			*Gal*/*CG9092*	Moderate (14)	50680 ^1^
type III (adult)					42922 ^1^
(b) GM2 gangliosidosis:					
Tay–Sachs disease (B variant)	*HEXA*	β-Hexosaminidase subunit α	*Hexo1*/ *CG1318*	Moderate (13)	67312 ^1^
			*Hexo2*/ *CG1787*	Moderate (12)	57199 ^1^
			*fdl*/*CG8824*	Moderate (11)	52987 ^1^
					28298 ^1^
Sandhoff disease (0 variant)	*HEXB*	β-Hexosaminidase subunit β	*Hexo1*/ *CG1318*	High (14)	67312 ^1^
			*Hexo2*/ *CG1787*	Moderate (12)	57199 ^1^
			*fdl*/*CG8824*	Moderate (12)	52987 ^1^
					28298 ^1^
(c) GM2 activator deficiency (AB variant)	*GM2A*	GM2 Ganglioside activator	-	-	-
G. Gaucher disease (GD)					
GD type 1	*GBA1*	β-Glucocerebrosidase 1/β-Glucosidase 1	*Gba1a*/ *CG31148*	High (15)	38379 ^1^
GD type 2					39064 ^1^
GD type 3			*Gba1b*/ *CG31414*	High (15)	38970 ^1^
Fetal/Perinatal lethal Gaucher disease					38977 ^1^
Atypical Gaucher disease due to Saposin C deficiency	*PSAP*	Prosaposin	*Sap-r*/ *CG12070*	High (14)	v51129 ^2^
					v51130 ^2^
Gaucher-like disease/ Gaucher disease- ophthalmoplegia-cardiovascular calcification syndrome/Gaucher disease type 3C	*GBA1*	β-Glucosylceramidase 1	*Gba1a*/ *CG31148*	High (15)	38379 ^1^
					39064 ^1^
			*Gba1b*/ *CG31414*	High (15)	38970 ^1^
					38977 ^1^
H. Globoid cell leukodystrophy— Krabbe disease	*GALC*	Galactosylceramidase	-	-	-
I. Lipid storage disease					
(a) Lysosomal acid lipase deficiency					
Cholesterol ester storage disease	*LIPA*	Lipase A lysosomal acid type, Cholesterol ester hydrolase	*Lip3*/*CG8823*	High (15)	65025 ^1^
Wolman disease					
(b) Niemann–Pick disease type C:					
type C1	*NPC1*	NPC Intracellular cholesterol transporter 1	*Npc1a*/ *CG5722*	High (16)	37504 ^1^
			*Npc1b*/ *CG12092*	Moderate (11)	38296 ^1^
			*SCAP*/ *CG33131*	Low (2)	31566 ^1^
type C2	*NPC2*	NPC Intracellular cholesterol transporter 2	*Npc2a*/ *CG7291*	High (16)	38237 ^1^
			*Npc2b*/ *CG3153*	Moderate (7)	38238 ^1^
					42914 ^1^
			*Npc2d*/ *CG12813*	Moderate (6)	v31095 ^2^
			*Npc2c*/ *CG3934*	Moderate (6)	61315 ^1^
			*Npc2e*/ *CG31410*	Moderate (6)	67956 ^1^
			*Npc2f*/ *CG6164*	Moderate (4)	v102172 ^2^
					v12915 ^2^
			*Npc2h*/ *CG11315*	Moderate (3)	67803 ^1^
			*Npc2g*/ *CG11314*	Moderate (3)	63030 ^1^
J. Metachromatic leukodystrophy (MLD)	*ASRA PSAP*	Arylsulfatase A Prosaposin			
K. Multiple Sulfatase deficiency (MSD)/Mucosulfatidosis	*SUMF1*	Sulfatase modifying factor 1, Formylglycine-generating enzyme	*CG7049*	High (14)	51896 ^1^
Action myoclonus-renal failure syndrome/Myoclonus-nephropathy syndrome/Progressive myoclonic epilepsy type 4	*SCARB2*	Scavenger receptor class B member 2, Lysosomal integral membrane protein II	*emp*/ *CG2727*	High (15)	40947 ^1^

^1^ Bloomington *Drosophila* Stock Center; ^2^ Vienna *Drosophila* Resource Center; N/A: Not Applicable.

**Table 3:** Summary of phenotypic defects in *Drosophila* models of LSDs. Overview of lifespan and locomotor phenotypes observed following RNAi-mediated knockdown of LSD-related genes in neuronal (*elav-GAL4*) or midgut (*NP1-GAL4*) tissues. Phenotype Severity Scale: (+++): severe reduction; (++): moderate reduction; (+): mild but significant reduction; (-): no significant difference; (n.d.): not determined (assay not performed). Sex Bias: indicates if the phenotype severity was significantly greater in one sex (*male-* or *female-biased*). “None” indicates comparable effects in both sexes.

Modeled Disease	Human Gene	*Drosophila* Ortholog	Neuronal Lifespan	Neuronal Climbing	Midgut Lifespan	Sex Bias
Gaucher	*GBA1*	*Gba1a*	+++	++	+	Male-biased
		*Gba1b*	+++	++	+	Male-biased
Fabry	*GLA*	*CG7997*	-	n.d.	-	None
		CG5731	+	++	+++	Female-biased
Niemann–Pick C	*NPC1*	*Npc1a*	+++	+++	++	Male-biased
	*NPC2*	*Npc2a*	+++	-	+	Male-biased
Tay–Sachs/ Sandhoff	*HEXA/* *HEXB*	*Hexo1*	-	n.d.	-	None
		*Hexo2*	++	n.d.	-	Male-biased
Pompe	*GAA*	*GCS2alpha*	+	n.d.	-	Male-biased
		*tobi*	-	n.d.	-	None
Hurler	*IDUA*	*Idua*	++	++	-	Male-biased
Hunter	*IDS*	*Ids*	+	n.d.	-	Male-biased
Sly	*GUSB*	*CG15117*	+	++	++	Male-biased

## Data Availability

The original contributions presented in this study are included in the Original Article/Supplementary Material. Further inquiries can be directed at the Corresponding Authors.

## Supplementary Materials

The following supporting information can be downloaded at: https://www.mdpi.com/article/10.3390/cells15010006/s1; Figure S1. Structural alignment of Gaucher disease-related proteins; Figure S2. Structural alignment of Fabry disease-related proteins; Figure S3. Structural alignment of Niemann–Pick disease type C1- and C2-related proteins; Figure S4. Structural alignment of Tay–Sachs and Sandhoff disease-associated proteins; Figure S5. Structural alignment of proteins related to Pompe disease; Figure S6. Structural alignment of Hurler syndrome-associated proteins; Figure S7. Structural alignment of Hunter syndrome-related proteins; Figure S8. Structural alignment of Sly disease-associated proteins; Figure S9. Locomotor deficits in *Drosophila* models of Gaucher disease; Figure S10. Genetic modeling of Fabry disease in the *Drosophila* brain–midgut axis, through targeting of the CG7997 ortholog gene in vivo.; Figure S11. In vivo genetic modeling of Fabry disease in *Drosophila* via *CG5731* targeting; Figure S12. Locomotor deficits in *Drosophila* models of Niemann–Pick disease type C; Figure S13. In vivo genetic modeling of Hurler disease in *Drosophila* via *Idua* targeting; Figure S14. In vivo genetic modeling of Sly disease in *Drosophila* via *CG15117* ortholog gene targeting; Table S1: The gene-specific DNA oligonucleotide primers used in this study.
