# Supplementary material for: Genetic Modeling of Lysosomal Storage Disorders (LSDs) in the Brain–Midgut Axis of Drosophila melanogaster During Aging
Source: Cells. 2025 Dec 19;15(1):6. doi: 10.3390/cells15010006 (PMC12786133; doi:10.3390/cells15010006)
Supplement: Supplementary file 1 [file cells-15-00006-s001.zip › cells-4023561-supplementary.pdf]

cells

MDPI

Article

Genetic Modeling of Lysosomal Storage Disorders (LSDs), in the Brain-Midgut Axis of *Drosophila melanogaster*, During Aging

Sophia P. Markaki, Nikoleta-Joana M. Kiose, Zoi A. Charitopoulou, Stylianos Kougioumtzoglou, Athanassios D. Velentzas*,# and Dimitrios J. Stravopodis*,#

Section of Cell Biology and Biophysics, Department of Biology, School of Science, National and Kapodistrian University of Athens (NKUA), 157 01, Athens, Greece (GR); smarkak@biol.uoa.gr (S.P.M.); nikoletajoanaq@gmail.com (N.-J.K.); zoecharitopoulou@gmail.com (Z.C.); steliosk7777@gmail.com (S.K.)

*These authors contributed equally to this work

#Corresponding authors: tveletz@biol.uoa.gr (A.D.V.); dstravop@biol.uoa.gr (D.J.S.)

Supplementary Material

Table S1. Gene-specific, DNA oligonucleotide, primers used in this study: Lysosomal Storage Disorder (LSD); *Drosophila* Gene Name; NCBI Reference Sequence; DNA Oligonucleotide Primer Sequence (F: Forward; R: Reverse); Primer Length (bp); and Product Length (bp), for the Human LSD and *Drosophila* homolog genes, herein, examined. bp: base pairs.

Lysosomal Storage Disorder (LSD)	*Drosophila* Gene Name	NCBI Reference Sequence	DNA Oligonucleotide Primer Sequence (5'→3')		Primer Length (bp)	Product Length (bp)
Gaucher disease	*Gba1a* / *CG31148*	NM_142915.3	F	AGGACATCACCCTGTTTGGC	20	78
			R	GTCGAGTGCGGTTCATCATTTT	22	
	Gba1b / *CG31414*	NM_001275951.1	F	CCCTGGCAGCAGGCTATCTG	20	111
			R	TGGGTAGGTGTACCGCTGAT	20	
Fabry disease	*CG7997*	NM_137275.4	F	ATTCAGTCGGTGCCACTAGC	20	142
			R	CCAGGACATCCAGCCCATAG	20	
	CG5731	NM_135510.3	F	TGGCTACAGAGTGGAGGACC	20	94
			R	CACGCCCGAAGGATTTACCT	20	
Niemann-Pick disease type C1	*Npc1a* / *CG5722*	NM_135513.5	F	GCGATAAGAATCAGGTGGAGC	21	133
			R	TCAGCCTGCTTTGGTGAACA	20	
Niemann-Pick disease type C2	*Npc2a* / *CG7291*	NM_134793.5	F	AACACCACGGTCAGCTTCTC	20	148
			R	GGCACTTCAGACCACTGTCC	20	
Tay-Sachs / Sandhoff disease(s)	*Hexo1* / *CG1318*	NM_079200.5	F	TGAACAGCAGCCAAGTTCAGT	21	95
			R	TCGTCAACCTCAGCACAGAAA	21	
	Hexo2 / *CG1787*	NM_080342.4	F	AGCAAGGATCAGGTTCTCGG	20	81
			R	CCAGATTCGGGATTCCAGTG	20	
Pompe disease	*GCS2alpha* / *CG14476*	NM_167758.2	F	GGTCACATTGCTTGGGTCCT	20	105
			R	CTTGTCCCGTGTTTCAAACTTTA	23	
	tobi / *CG11909*	NM_143134.3	F	CGCTGTGGATTCTGGTGAAC	20	99
			R	TCGCCCAAAAGGAACTCATCAT	22	
Hurler syndrome		NM_135645.2	F	GGAGTGGATGGGCAATCTGG	20	123

	Idua / *CG6201*		R	AATCTGGAGGAACCGTGTCG	20	
Hunter syndrome	*Ids* / *CG12014*	NM_139557.2	F	GGAATGCTGCCCACAAAGC	19	150
			R	CACATCGTGCCAATCTCTGCT	21	
Sly disease	*CG15117*	NM_166324.2	F	TGCGGATTCGTACTTTGAATTAG	23	149
			R	ACCAGGCATTGTAGCGATTG	20	
Endogenous control	*Act5C* / *CG4027*	NM_001014725.2	F	GATCGGGATGGTCTTGATTC	20	149
			R	GTGGTTCCGCTCTTTTCATC	20	

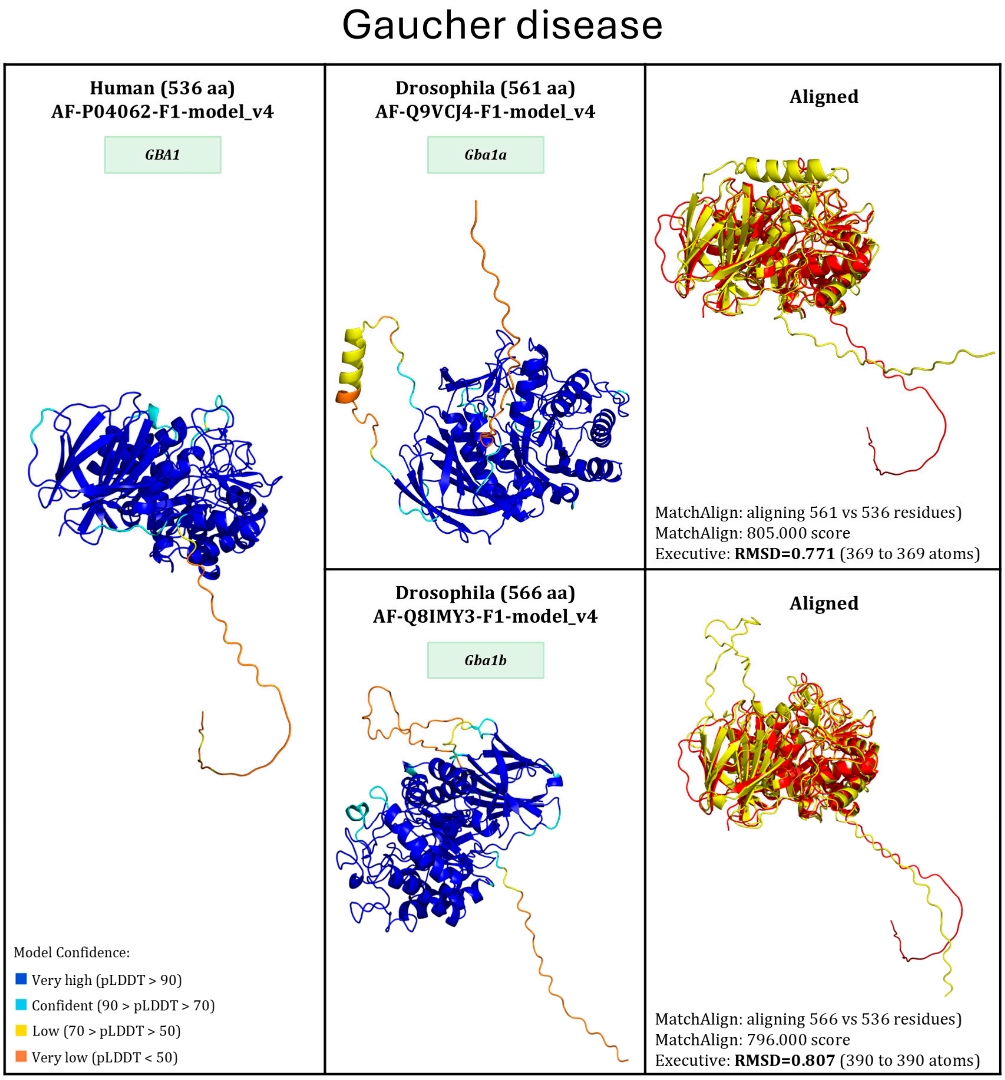

Figure S1. Structural alignment of Gaucher disease-related proteins. AlphaFold-predicted structures of proteins derived from the Human *GBA1* (left panel) and its *Drosophila* ortholog *Gba1a* and *Gba1b* (middle panels) genes are shown. Structural alignments between the Human protein (red) and each *Drosophila* counterpart (yellow), being generated and visualized using the PyMOL molecular graphics system, are presented in the right panels. The high degree of structural similarity observed (RMSD values ≤ 0.807 Å) supports the evolutionary conservation and functional relevance of these fly proteins as molecular targets for Gaucher-disease genetic modeling in *Drosophila*.

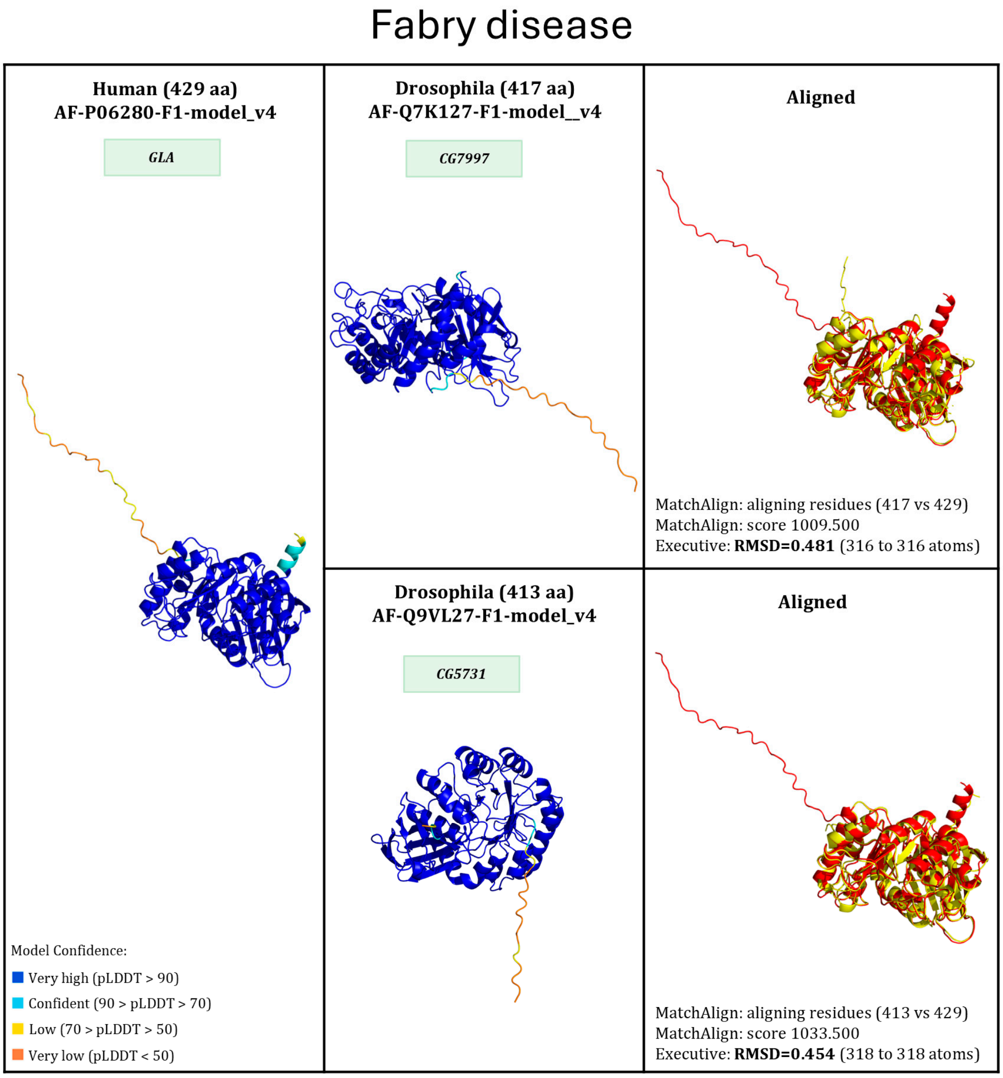

Figure S2. Structural alignment of Fabry disease-related proteins. AlphaFold-predicted structures of proteins derived from the Human *GLA* gene (left panel) and its *Drosophila* ortholog *CG7997* and *CG5731* (middle panels) genes are illustrated. Structural alignments between the human (red) and each *Drosophila* protein (yellow), being aligned and visualized using the PyMOL molecular graphics system, are presented in the right panels. The alignments demonstrate very strong structural conservation, with obtained RMSD values of 0.481 Å for GLA-CG799 and 0.454 Å for GLA-CG5731 highlighting the downregulation of these fly gene-encoding proteins as powerful and reliable tools for genetically modeling Fabry disease-related pathologies in *Drosophila*.

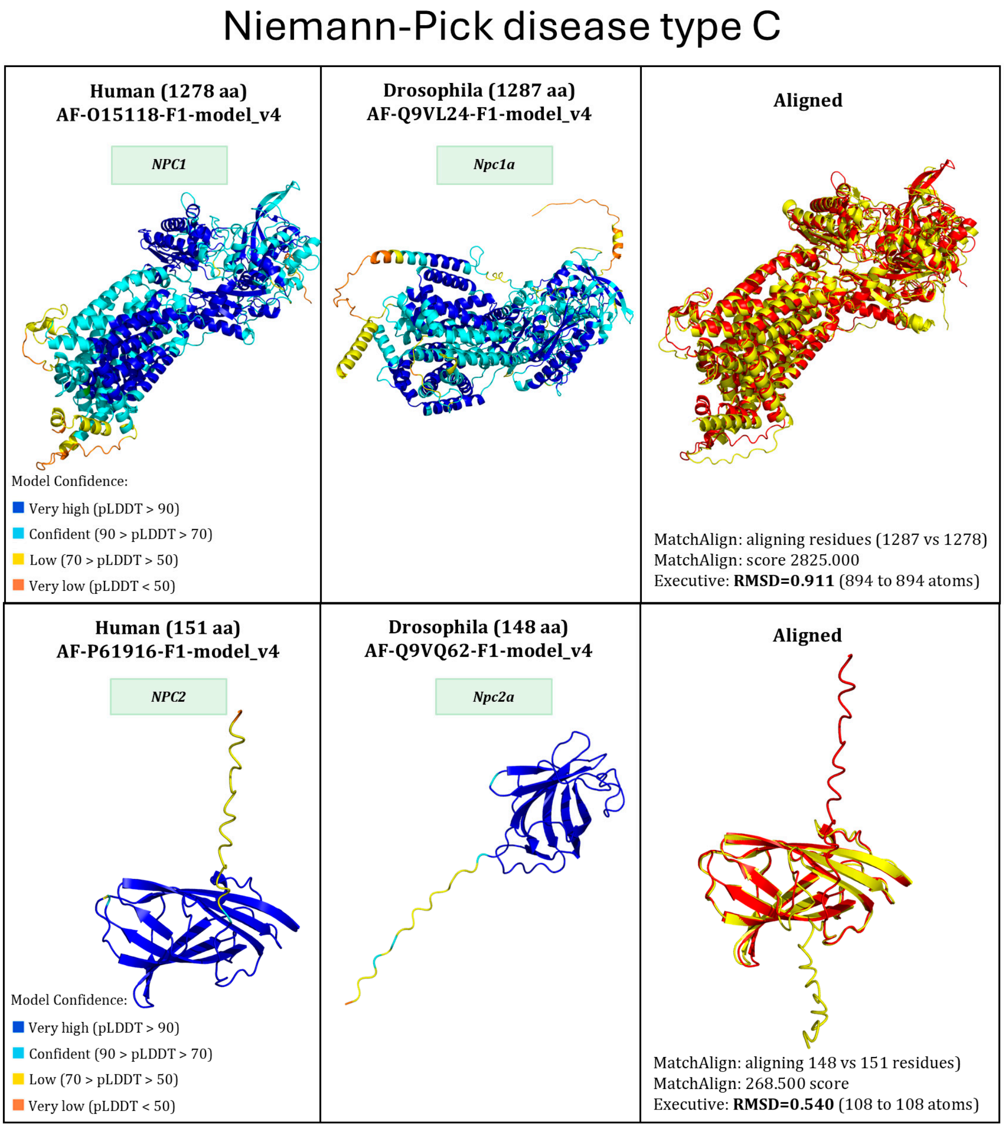

Figure S3. Structural alignment of Niemann-Pick disease type C1- and C2-related proteins. AlphaFold-predicted structures of proteins derived from the Human *NPC1* and *NPC2* (left panels), and their respective *Drosophila* ortholog *Npc1a* and *Npc2a* (middle panels) genes are shown. Structural alignments between each Human protein (red) and its *Drosophila* counterpart (yellow), being generated and visualized using the PyMOL molecular graphics system, are presented in the right panels. The high degree of structural similarity observed supports the evolutionary conservation and functional relevance of these fly gene products as molecular targets for genetic modeling of Niemann-Pick disease type C in *Drosophila*.

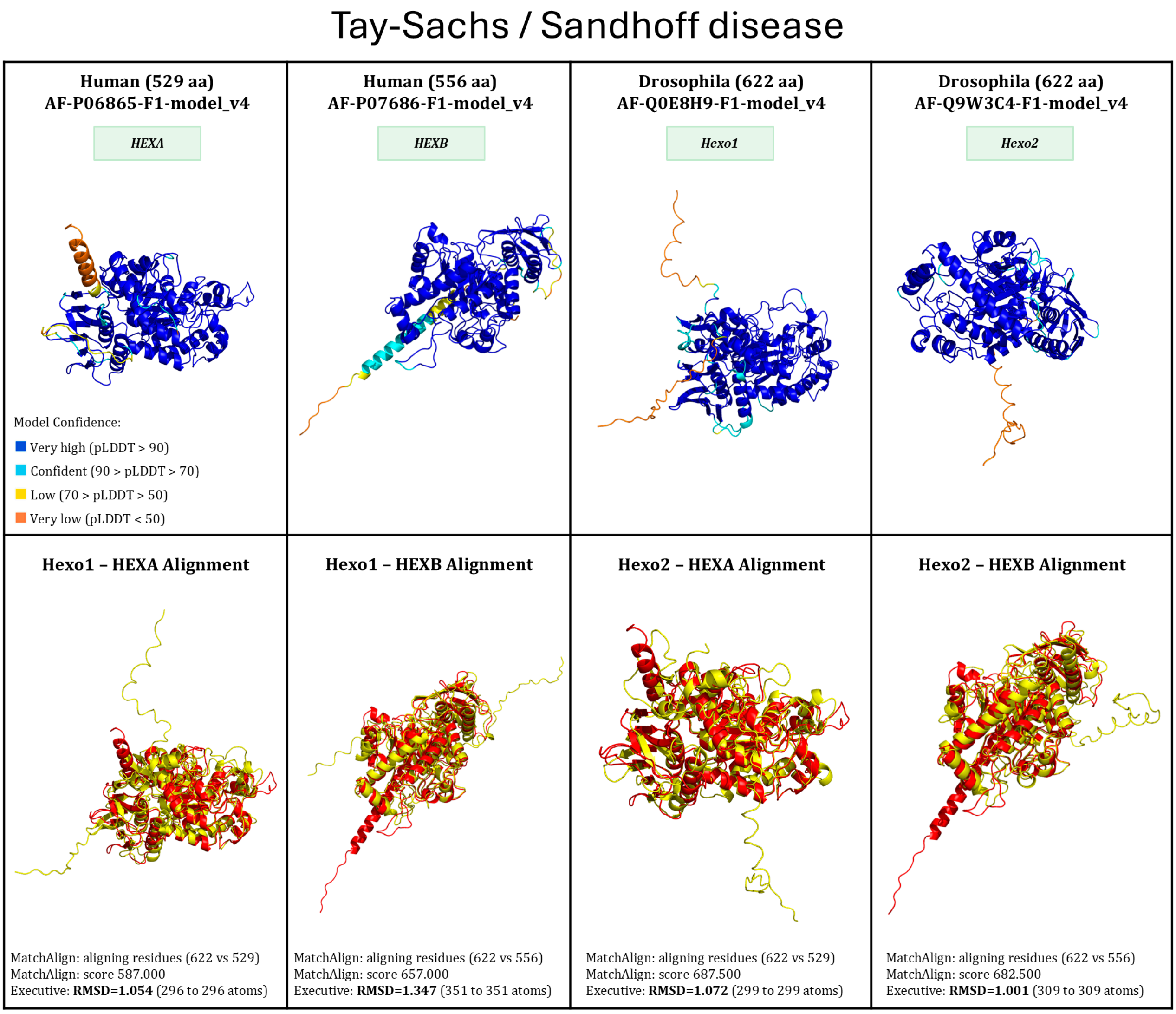

Figure S4. Structural alignment of Tay-Sachs and Sandhoff disease-associated proteins. AlphaFold-predicted structures of proteins derived from the Human genes *HEXA* and *HEXB*, along with their *Drosophila* orthologs *Hexo1* and *Hexo2*, are illustrated in the upper panels. Structural alignments between Human proteins (red) and their respective *Drosophila* counterparts (yellow), being generated and visualized using the PyMOL molecular graphics system, are presented in the lower panels. Structural similarities observed dictate the capacity of fly proteins to serve as molecular targets for genetic modeling of Tay-Sachs- and Sandhoff-disease pathologies in *Drosophila*.

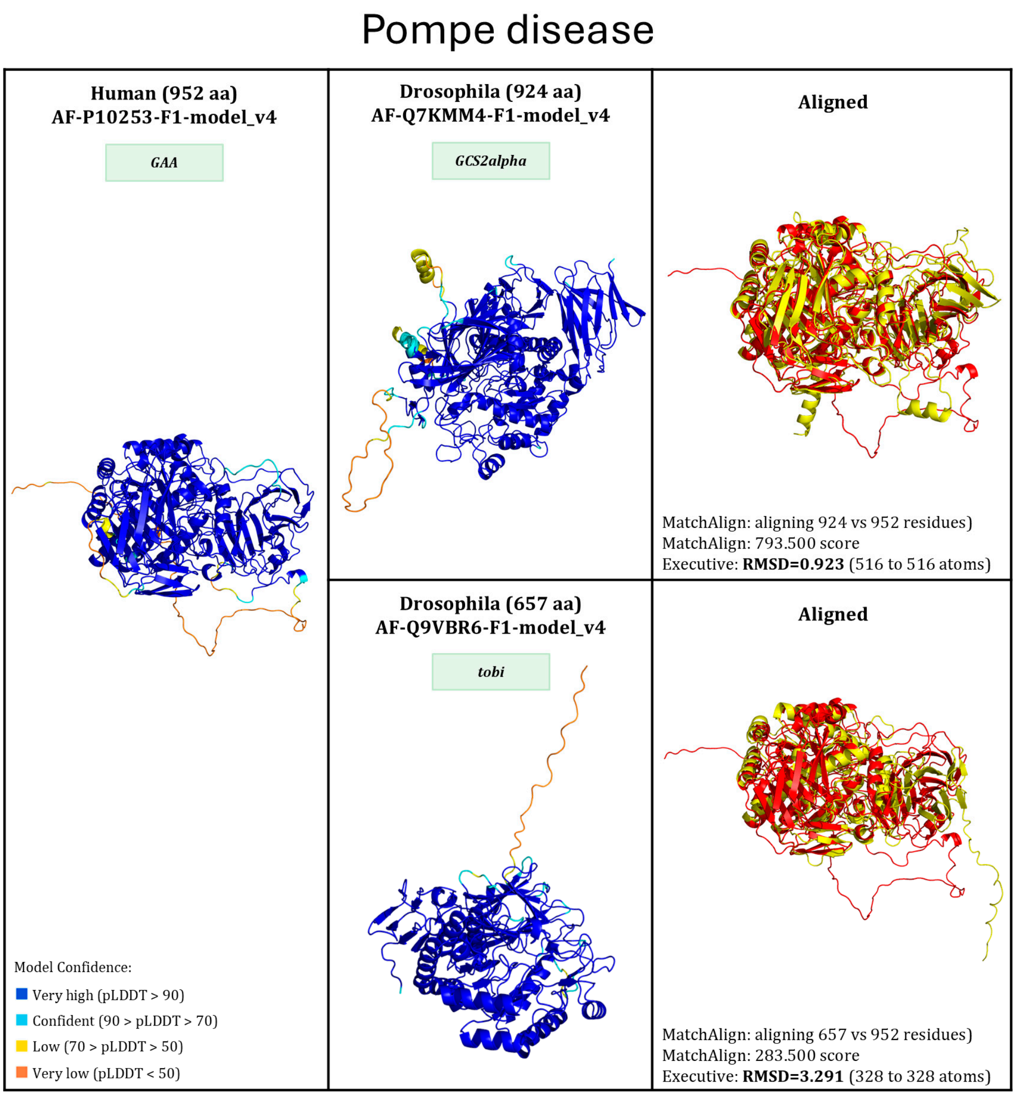

Figure S5. Structural alignment of proteins related to Pompe disease. The left panel illustrates the AlphaFold-predicted structure of the protein that derives from the Human *GAA* gene. The middle panels illustrate the structures of proteins derived from the respective *Drosophila* ortholog, *GCS2a* and *tobi*, genes. The right panels present structural alignments between the Human protein (red) and each *Drosophila* counterpart (yellow), being generated and visualized using the PyMOL molecular graphics system. Structural similarities unveiled indicate the proficiency of fly proteins to serve as molecular targets for genetic modeling of Pompe-disease pathology in *Drosophila*.

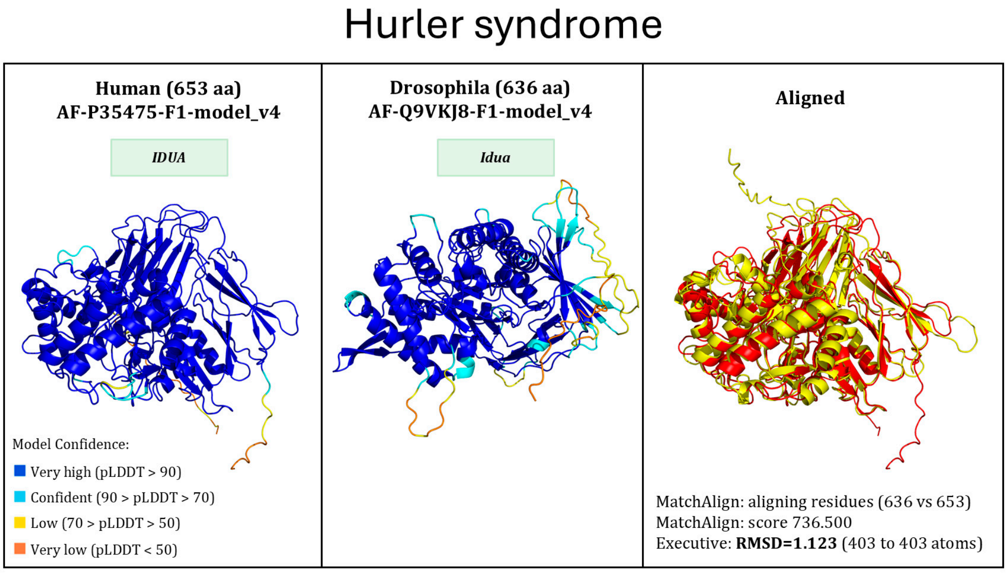

Figure S6. Structural alignment of Hurler syndrome-associated proteins. AlphaFold-predicted structures of proteins derived from the Human gene *IDUA* (left panel) and its *Drosophila* ortholog *Idua* (middle panel) are shown. The right panel illustrates the structural alignment performed using PyMOL, with the Human protein colored red and the *Drosophila* protein colored yellow. Structural similarities observed dictate the capacity of fly proteins to serve as molecular targets for the genetic modeling of Hurler-syndrome pathology in *Drosophila*.

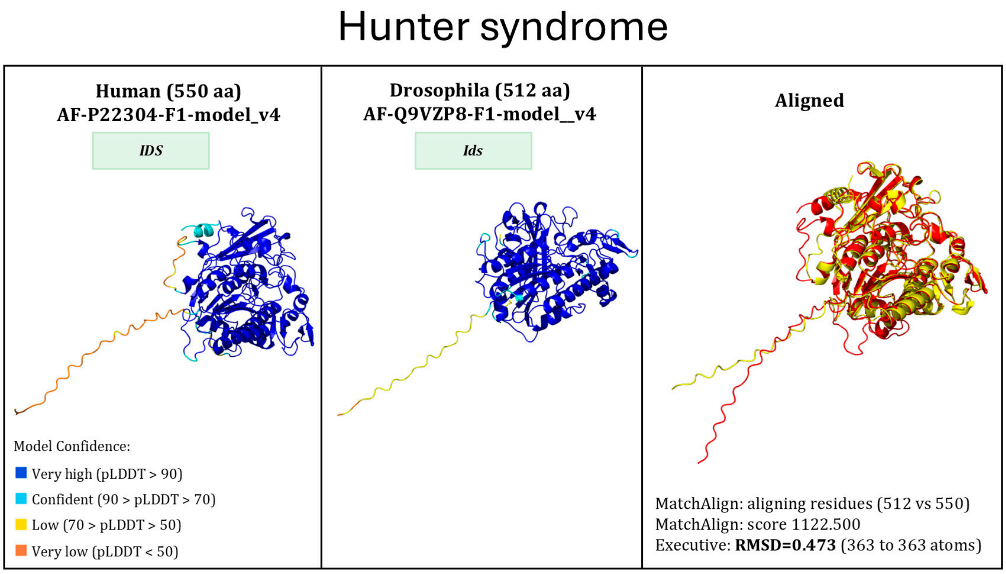

Figure S7. Structural alignment of Hunter syndrome-related proteins. AlphaFold-predicted structures of proteins derived from the Human gene *IDS* (left panel) and its *Drosophila* ortholog *Ids* (middle panel) are illustrated. The right panel illustrates the structural alignment performed using PyMOL, with the Human protein colored red and the *Drosophila* protein colored yellow. The alignment reveals a high degree of structural conservation, with an RMSD value of 0.473 Å, supporting the capacity of *Drosophila* ortholog to serve as molecular target for genetic modeling of Hunter-syndrome pathology in *Drosophila*.

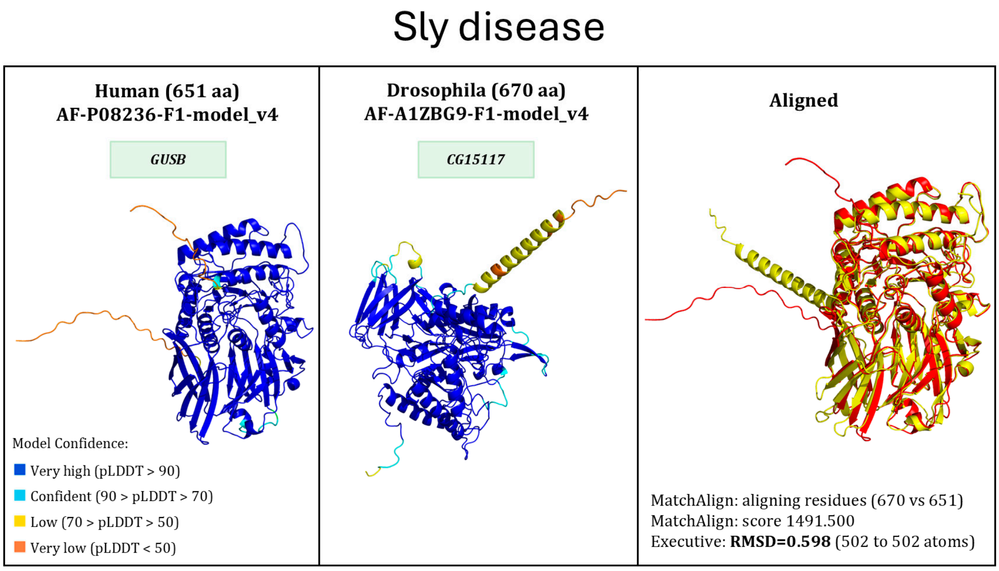

Figure S8. Structural alignment of Sly disease-associated proteins. AlphaFold-predicted structures of proteins derived from the Human *GUSB* gene (left panel) and its *Drosophila* ortholog *CG15117* (middle panel) are shown. The right panel illustrates the structural alignment performed using PyMOL, with the Human protein colored red and the *Drosophila* protein colored yellow. The high degree of structural similarity observed (RMSD = 0.598 Å) underscores the evolutionary conservation and supports the functional relevance of the fly protein as a critical molecular target for genetic modeling of Sly disease-related pathology in *Drosophila*.

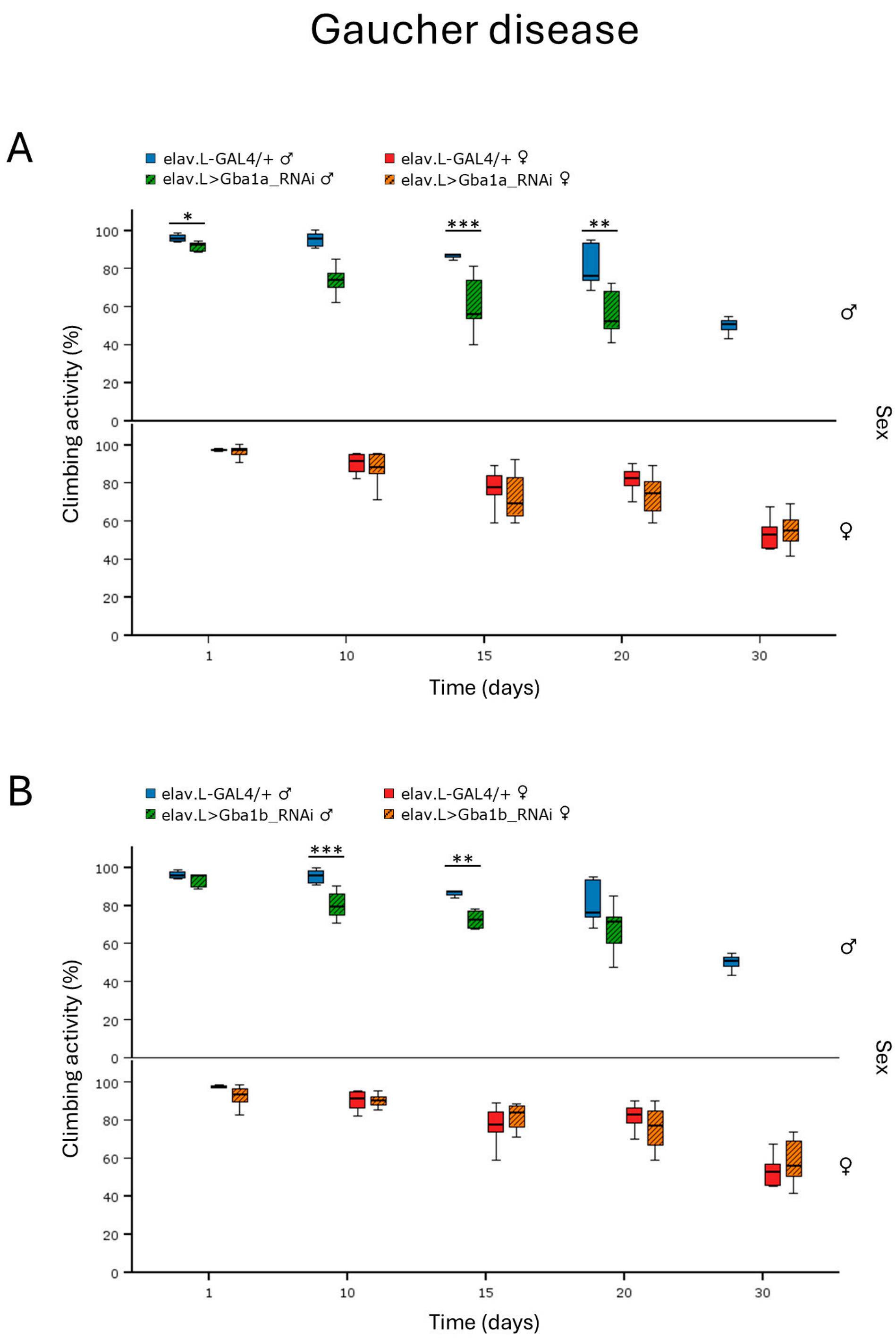

Figure S9. Locomotor deficits in *Drosophila* models of Gaucher disease. Assessment of climbing performance (negative geotaxis assay) in male and female flies, following neuronal-specific (brain) knockdown of (**A**) *Gba1a* and (**B**) *Gba1b* gene, during aging (0–30 days, post-eclosion). Results are presented as “box-and-whisker” plots. Statistical significance is indicated as: *$p < 0.05$, **$p < 0.01$ and ***$p < 0.001$. Note: Statistical analysis was not performed at day 30 for male experimental groups, due to insufficient sample size caused by high mortality. Sample sizes: n > 300 flies per genotype, time point and sex (pooled from 3 independent biological replicates, with n > 100 per replicate), except whereat noted due to attrition.

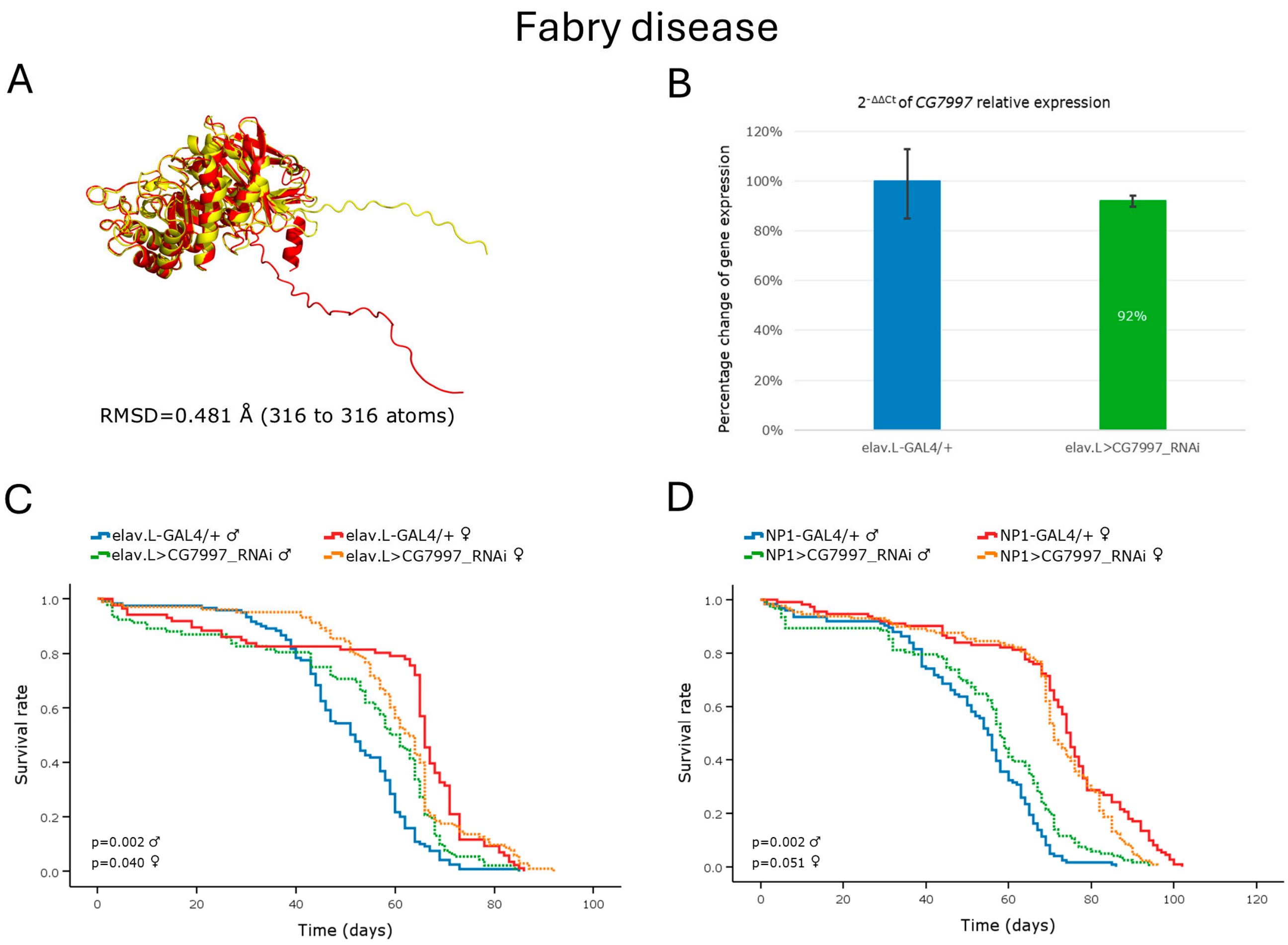

Figure S10. Genetic modeling of Fabry disease in *Drosophila* brain-midgut axis, through targeting the CG7997 ortholog gene, *in vivo*. (**A**) PyMOL-mediated structural alignment of the AlphaFold-generated protein structure of the human *GLA* gene (red) aligned to its *Drosophila* counterpart that is being encoded by the *CG7997* ortholog (yellow). (**B**) Relative expression levels of the *CG7997* gene in neuronal (brain) tissues of RNAi-targeted flies (elav.L>CG7997_RNAi), compared to control (elav.L-GAL4/+) population, assessed by RT-qPCR. (**C**) Kaplan-Meier survival curves of male and female flies following neuronal-specific (*elav-GAL4*) knockdown of *CG7997*. (**D**) Lifespan profiles of male and female flies, after *CG7997* gene silencing, specifically in midgut tissues (NP1>CG7997_RNAi). Sample sizes: total n > 300 flies per genotype and sex (pooled from 3 independent biological replicates, with n > 100 per replicate).

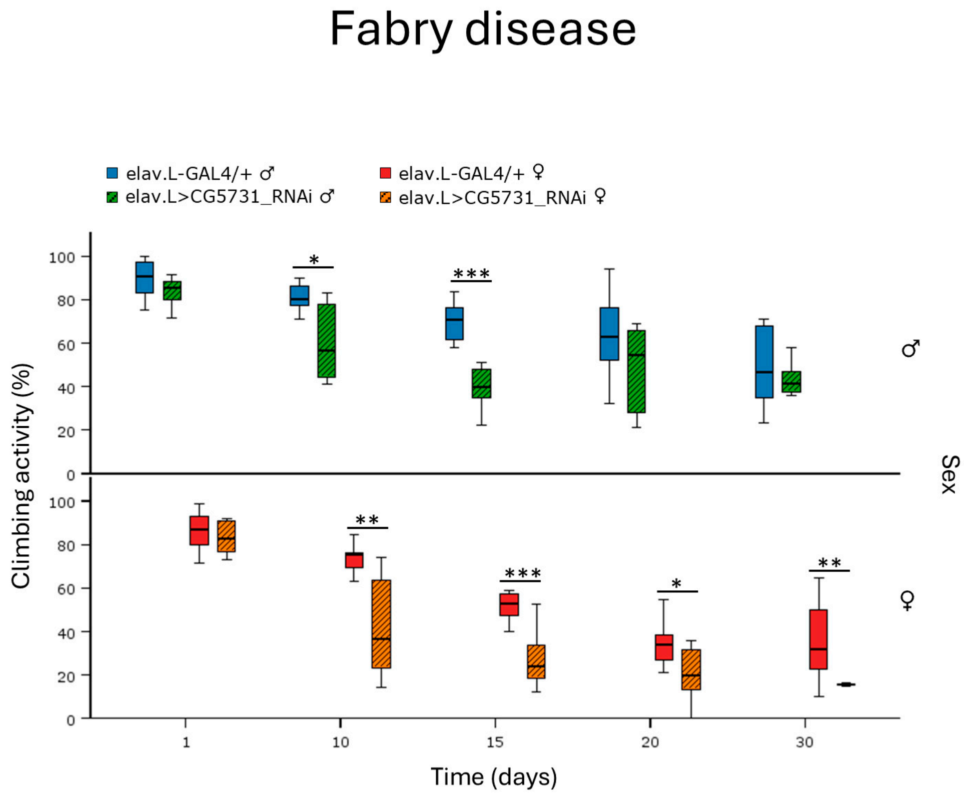

Figure S11. *In vivo* genetic modeling of Fabry disease in *Drosophila* via *CG5731* targeting. Assessment of climbing performance (negative geotaxis assay), during aging (0-30 days, post-eclosion), demonstrating progressive locomotor decline in flies with neuronal (brain)-specific *CG5731* downregulation, compared to control fly population. Results are presented as "box-and-whisker" plots. Statistical significance is indicated as: *$p < 0.05$, **$p < 0.01$ and ***$p < 0.001$. Sample sizes: n > 300 flies per genotype, time point and sex (pooled from 3 independent biological replicates, with n > 100 per replicate).

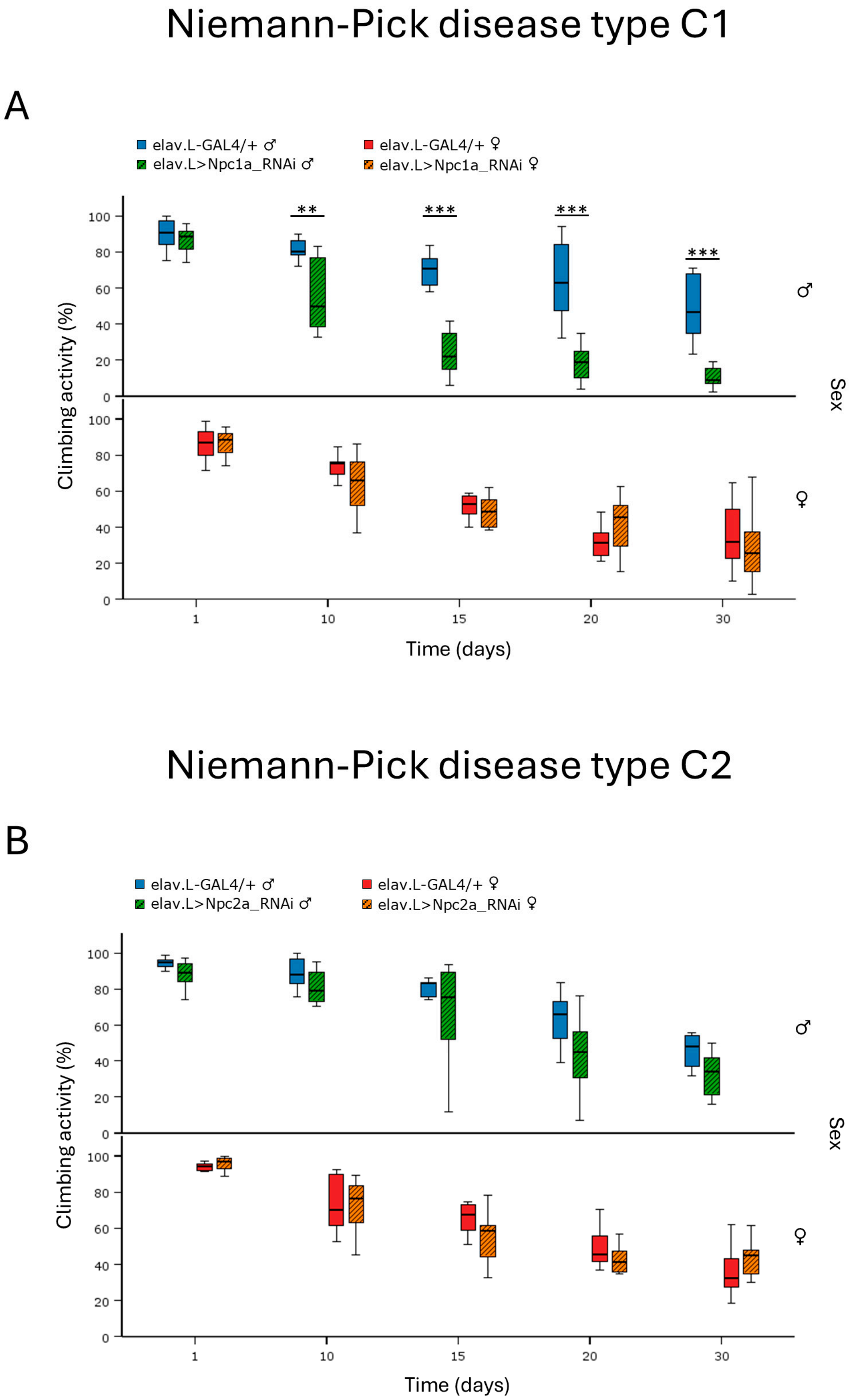

Figure S12. Locomotor deficits in *Drosophila* models of Niemann-Pick disease type C. Assessment of climbing performance (negative geotaxis assay) in male and female flies following neuronal-specific (brain) knockdown of (**A**) *Npc1a* and (**B**) *Npc2a* gene, as a function of aging (0–30 days, post-eclosion). Results are presented as "box-and-whisker" plots. Statistical significance is indicated as: $^{**}p < 0.01$ and $^{***}p < 0.001$. Sample sizes: n > 300 flies per genotype, sex and time point (pooled from 3 independent biological replicates, with n > 100 per replicate).

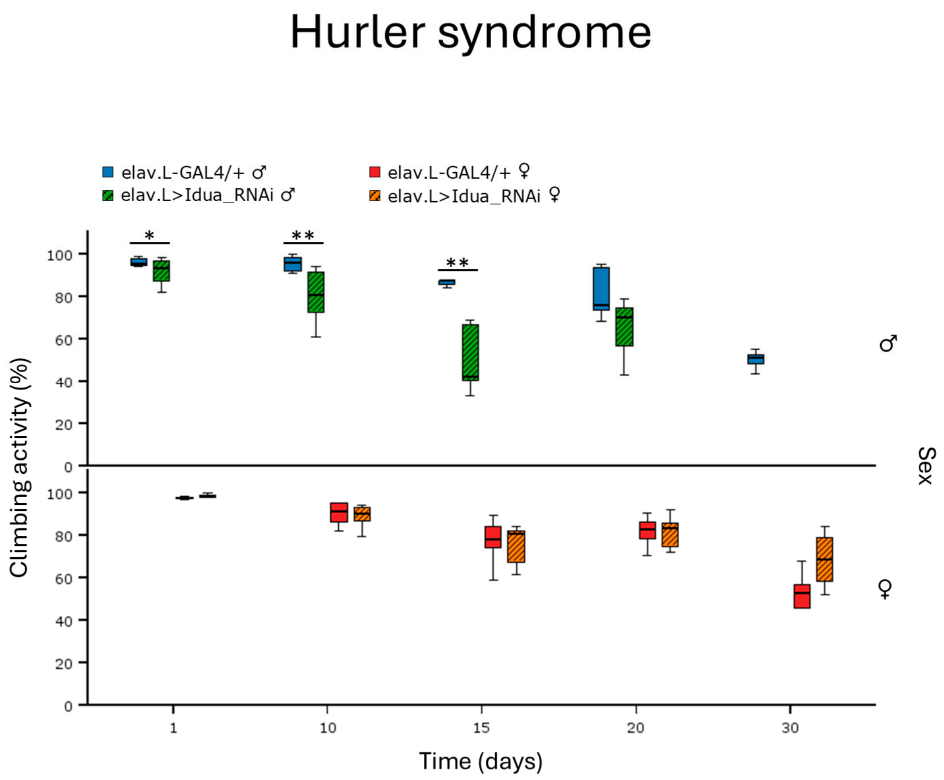

Figure S13. *In vivo* genetic modeling of Hurler disease in *Drosophila* via *Idua* targeting. Assessment of climbing performance (negative geotaxis assay), in male and female flies, following neuronal-specific (brain) knockdown of *Idua* gene, as a function of age (1–30 days, post-eclosion). Results are presented as "box-and-whisker" plots. Statistical significance is indicated as: **p* < 0.05 and ***p* < 0.01. Note: statistical analysis was not performed at day 30 for male experimental groups, due to insufficient sample size caused by high mortality. Sample sizes: n > 300 flies per genotype, time point and sex (pooled from 3 independent biological replicates, with n > 100 per replicate), except whereat noted due to attrition.

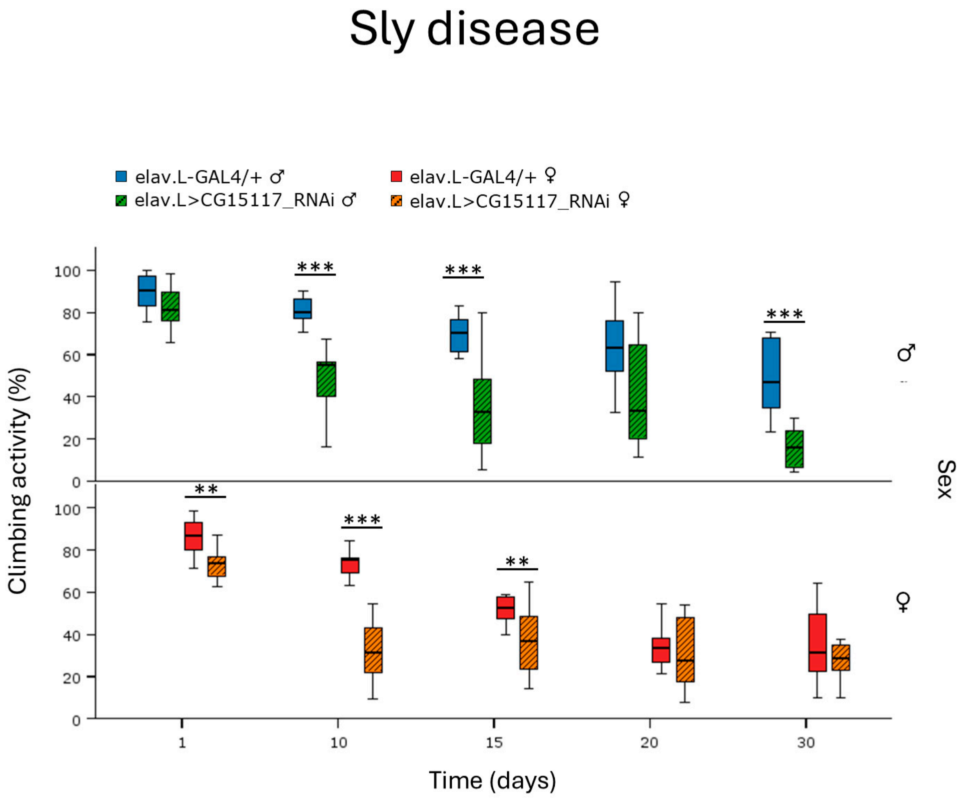

Figure S14. *In vivo* genetic modeling of Sly disease in *Drosophila* via *CG15117* ortholog gene targeting. Climbing performance of transgenic flies with neuronal (brain)-specific *CG15117*-gene downregulation (elav.L>CG15117_RNAi), versus control genetic settings (elav.L-GAL4/+), measured and quantified over time (0-30 days, post-eclosion) (using the negative-geotaxis assay), demonstrating the progressive, age- and sex-dependent, impairment in motor function(s). Results are presented as "box-and-whisker" plots. Statistical significance is indicated as: $^{**}p < 0.01$ and $^{***}p < 0.001$. Sample sizes: n > 300 flies per genotype, sex and time point (pooled from 3 independent biological replicates, with n > 100 per replicate).
